# Analytical solution for MHD nanofluid flow over a porous wedge with melting heat transfer

**DOI:** 10.1016/j.heliyon.2024.e34888

**Published:** 2024-07-22

**Authors:** Ali Ahmadi Azar, Payam Jalili, Zahra Poolaei Moziraji, Bahram Jalili, Davood Domiri Ganji

**Affiliations:** a Department of Mechanical Engineering, North Tehran Branch, Islamic Azad University, Tehran, Iran; b Department of Mechanical Engineering, Babol Noshirvani University of Technology, P.O. Box 484, Babol, Iran

**Keywords:** Hybrid analytical and numerical method, Magnetohydrodynamic flow, Permeable wedge, Porous medium, Hyperbolic tangent nanofluid, Time-independent

## Abstract

This study employs the Hybrid Analytical-Numerical (HAN) method to investigate steady two-dimensional magnetohydrodynamic (MHD) nanofluid flow over a permeable wedge. Analyzing hyperbolic tangent nanofluid flow, the governing time-independent partial differential equations (PDEs) for continuity, momentum, energy, and concentration transform into a set of nonlinear third-order coupled ordinary differential equations (ODEs) through similarity transformations. These ODEs encompass critical parameters such as Lewis and Prandtl numbers, Brownian diffusion, Weissenberg number, thermophoresis, Dufour and Soret numbers, magnetic field strength, thermal radiation, power law index, and medium permeability. The study explores how variations in these parameters impact the velocity field, skin friction coefficient, Nusselt, and Sherwood numbers. Noteworthy findings include the sensitivity of fluid velocity to parameters like Weissenberg number, power law index, wedge angle, magnetic field strength, permeability, and melting heat transfer. The skin friction coefficient experiences a significant increase with specific parameter changes, while Nusselt and Sherwood numbers remain relatively constant. The local Reynolds number significantly affects Nusselt and Sherwood numbers, with a less pronounced impact on the skin friction coefficient. The study's uniqueness lies in employing the analytical HAN method and extracting recent insights from the results.

**Table:** Nomenclature

Cartesian coordinate components (m)	(x,y)	Electrical conductivity of the nanofluid (S/m)	σ
Cartesian velocity components (m/s)	(u,v)	Heat capacity of nanofluid to normal fluid (−)	Λ
Velocity field along x-axis (m/s)	u(x,y)	Viscosity of nanofluid (kg/m.s)	ν
Velocity field along y-axis (m/s)	v(x,y)	Ratio of thermal diffusion (−)	$\kappa_{T}$
Temperature in dimensional form (K)	T,T(x,y)	Thermal conductivity of the nanofluid (W/m.K)	k
Concentration in dimensional form (mol/ $m^{3}$)	C,C(x,y)	The concentration of the nanofluid at the surface of the wedge (mol/ $m^{3}$)	$C_{w}$
Latent heat of the nanofluid (J/kg)	λ	Heat capacity of the solid surface (J/K)	$C_{s}$
Temperature of the nanofluid at far away from wedge(K)	$T_{\infty }$	The concentration of the nanofluid at far away from the solid surface of the wedge (mol/ $m^{3}$)	$C_{\infty }$
Temperature of the molten material from the surface of the wedge (K)	$T_{m}$	Density of solid surface (wedge) ($\text{kg}/m^{3}$)	ρ
Temperature of the solid material from the surface of the wedge (K)	$T_{0}$	Radiation heat flux ($W/m^{2}$)	$q_{r}$
Velocity distribution of the nanofluid outside of the boundary layer (m/s)	U(x)	Stefan-Boltzmann constant ($W/m^{2}K^{4}$)	$\sigma^{*}$
Positive real constant (−)	a	Absorption constant ($K^{4}/m$)	$\kappa^{*}$
Function of the magnetic field perpendicular to the wedge walls (T)	B(x)	Stream function (kg/m.s)	ψ,ψ(x,y)
constant of the magnetic field perpendicular to the wedge walls (−)	$B_{0}$	Dimensionless independent variable (−)	ξ
Permeability function for the porous wedge ($m^{2}\;\text{or}\;d$)	K(x)	Dimensionless velocity quantity (−)	$f^{\prime }\left(\xi \right)$
Constant of permeability for the porous wedge (−)	$K_{0}$	Dimensionless temperature quantity (−)	θ(ξ)
Wedge angle parameter (−)	m	Dimensionless concentration quantity (−)	ϕ(ξ)
Hartree pressure gradient (rad)	$\beta_{1}$	Weissenberg number (−)	Wi
The angle of the wedge (rad)	Ω	Permeability of the medium (−)	$K_{p}$
Stress tensor ($N/m^{2}\;\text{or}\;\text{Pa}$)	τ	Magnetic field parameter (−)	M
Power law index (−)	n	Thermal radiation parameter (−)	Rd
Null shear rate viscosity (PasorPa.s)	$\mu_{0}$	Prandtl number (−)	Pr
Infinite shear rate viscosity (PasorPa.s)	$\mu_{\infty }$	Dufour number (−)	$D_{4}$
The material constant of time (1/s)	Γ	Brownian diffusion parameter (−)	Nb
Transpose of a matrix (−)	T	Thermophoresis parameter (−)	Nt
Trace of a matrix (−)	tr	Lewis number (−)	Le
Second-order strain rate tensor invariant ($s^{-1}$)	A	Soret number (−)	Sr
Velocity vector (m/s)	V	The melting effect (−)	B
Gradient (−)	∇	Skin friction coefficient (−)	$C_{f}$
Thermophoresis diffusion	$D_{T}$	Nusselt number (−)	Nu
Brownian diffusion	$D_{B}$	Sherwood number (−)	Sh
Thermal diffusivity of nanofluid ($m^{2}/s$)	α	Local Reynolds number (−)	${Re}_{x}$
The density of solid nanoparticle ($\text{kg}/m^{3}$)	$\rho_{p}$	Specific heat of solid nanoparticles (J/Kg.K)	$c_{p}$
The density of base fluid ($\text{kg}/m^{3}$)	$\rho_{f}$	Specific heat of base fluid (J/Kg.K)	$c_{f}$

## 1 Introduction

### 1.1 Significance of the current study

Hyperbolic tangent fluid is a non-Newtonian fluid that shows shear thinning behavior. Among various non-Newtonian fluids, hyperbolic tangent fluid has several unique advantages due to its ease of computation, understanding, and strong physical clarifications. This fluid type can be found in various everyday products such as ketchup, lava, whipped cream, paints, and even blood. Researchers worldwide have conducted extensive research on hyperbolic tangent fluids under various conditions, which has led to significant advancements in this field. Magnetohydrodynamics (MHD) is a subset of fluid dynamics focused on the behavior of electrically conducting fluids when subjected to a magnetic field. It has important applications in power generation, plasma physics, and astrophysics. Nanofluids are fluids containing nanoparticles, proposed by Choi and Eastman [1] to enhance heat transfer. Nanofluids have widespread uses in various industries, including transportation, nuclear reactors, biomedicine, and electronics. Industrial and technological applications worldwide are significantly influenced by the combined effects of heat and mass transfer through a porous medium. Thermal radiation is a crucial aspect of current technology and industry, which is detected as heat or light and varies with the temperature of the surrounding environment. The study of the flow past wedge-shaped bodies has been a subject of significant interest among researchers worldwide due to its vast applications in engineering, science, technology, and other fields. The wedge problems mainly deal with the flows that are non-parallel to the plates. These challenges were initially characterized by the Falkner-Skan equation, a formulation that delineates the external flow patterns of laminar boundary layers. A substance that comprises a solid framework with interconnected empty spaces is commonly referred to as a porous medium. Examples of natural and artificial porous media include sponges, cement, soil, biological tissues, and bones. The porous structure of such materials enables the flow of fluids through them, and the properties of the empty spaces vary depending on the structure, size, shape, porosity, and composition of the material.

### 1.2 Literature review

Choi and Eastman [1] established that nanofluids significantly enhance heat transfer coefficients compared to base fluids, finding applications in diverse fields such as heat transfer, electronics, industrial processes, transportation, nuclear reactors, biomedicine, food, and detergents. Mustafa et al. [2] investigated heat and mass transfer in a viscous fluid between parallel plates, simplifying governing equations and employing the Homotopy Analysis Method (HAM) for a series solution. Environmental concerns and depletion of fossil fuels drive the exploration of alternative energy sources like solar energy. Mahian et al. [3] addressed this by studying nanofluids in solar thermal engineering systems. Using scattered literature sources, Lomascolo et al. [4] examined nanofluid heat transfer. Ibrahim [5] explored the impact of thermal radiation on the magnetohydrodynamic flow of a tangent hyperbolic fluid with nanoparticles on an enlarging sheet. The numerical analysis considered factors like Weissenberg number, slip parameters, power-law index, radiation parameter, thermophoresis parameter, and Biot number.

In their investigation of constrained tangent hyperbolic fluid flow on a stretched sheet, Ali et al. [6] took into account mixed convection, magnetohydrodynamics, and variable thermal conductivity. The effects of thermal radiation and changing thermal conductivity on magnetohydrodynamic tangent hyperbolic fluid flow with nanoparticles traveling through a stretched sheet were investigated by Atif et al. [7]. Using similarity conversion and the Shooting approach, Patil et al. [8] investigated steady-state magnetohydrodynamic boundary layer flow of tangent hyperbolic fluid across an exponentially stretched surface with heat source and chemical reaction. Sarkar and Endalew [9] examined thermophoresis and Brownian motion while examining the impact of permeability and the melting process on the hydromagnetic wedge flow of a Casson nanofluid. The effects of an angled magnetic field on the mass transfer flow and unsteady natural convection heat from an oscillating inclined plate were investigated by Endalew et al. [10]. Ibrahim and Gizewu [11] examined how a non-Newtonian fluid containing nanoparticles behaved as it passed by a stretched surface that had been unevenly thickened. Using the HAM, Kebede et al. [12] investigated heat and mass transport in a boundary layer flow of a nanofluid across a permeable stretching wedge. The flow of an electrically conducting viscous fluid across a curved surface with second-order slip was studied by Muhammad et al. [13], taking into account Joule heating, thermal radiation, and viscous dissipation. In a one-way extending surface whirling flow, Ramaiah et al. [14] investigated the effects of heat transfer and the Maxwell fluid mass phenomena, taking into account the magnetic field and the non-Fourier Cattaneo-Christov heat flux model. Heat and mass transport in a mixed convective flow of electrically conducting nanofluid via a thin Riga plate was studied by Vaidya et al. [15].

In a study by Azeem Khan et al. [16], the heat and mass transfer mechanisms of incompressible Sutterby nanofluid over a stretching non-porous surface were investigated, considering the effects of a magnetic field, Brownian motion, and thermophoresis. Using the similarity transformation method, the problem was transformed into a set of coupled ODEs, which were then solved numerically using the efficient "bvp4c" algorithm. The results suggested that Brownian motion increased the dimensionless thermal distribution, while the concentration field showed the opposite effect. Additionally, the instability parameter of the functions reduced the velocity and temperature field. Nanofluids have a wide range of engineering applications and are commonly found in our daily lives due to their ability to enhance energy efficiency and heat transfer in various thermal systems. Combining nanotechnology and thermal performance has presented numerous opportunities for scientists and researchers worldwide. In a recent study by Hussain and Azeem Khan [17], they examined the time-dependent Sutterby nanofluid, taking into consideration thermal radiation, stratification phenomenon, activation energy, and heat source-sink. To simplify PDEs into nonlinear dimensionless ODEs, they utilized similarity transformations. The bvp4c scheme numerical method was then employed to solve the nonlinear ODEs. With the use of tables and graphs, the following factors were carefully examined: temperature, concentration profiles, skin friction parameters, Nusselt number, and Sherwood number. According to the findings, the concentration profile falls as the Schmidt number rises, and the temperature profile rises with the thermal Biot number. Tabrez and Azeem Khan [18] conducted a study analyzing the behavior of Sutterby fluid with ferromagnetic properties. Their focus was on the impact of nanomaterials, including Brownian motion and viscous dissipations, on Sutterby nanofluid. Additionally, they explored the magnetic dipole effect, which has practical applications in bioengineering and engineering. The researchers utilized similarity transformations to generate the necessary ODEs, which were then solved utilizing the bvp4c technique. They visually analyzed the physical parameters involved and discussed their unique features. The results of the study indicated that the Brownian motion and thermophoretic influences had opposing effects on the nanofluid concentration. The velocity, thermal gradients, and concentration were all analyzed visually. They compared their findings to those of previous studies and found that they agreed. Rehman et al. [19] studied the chemically reactive process of time-dependent bioconvective magnetohydrodynamic Williamson nanofluid flowing toward a wedge with radiative heat, considering gyrotactic motile microorganisms. The main goal was to enhance heat transport. The study addressed partial differential equations (PDEs) by transforming them into ordinary differential equations (ODEs) using suitable modifications. Numerical solutions via the Runga-Kutta fourth-order method reveal key characteristics of nanoparticles, including liquid velocity, concentricity, thermal effects, drag friction factor, motile organisms, and heat and mass transport. Rehman et al. [20] studied melting and nonlinear mixed convection in unsteady Falkner-Skan wedge flow of Reiner-Philippoff nanofluid. The study considered thermally radiative effects, nanoparticle mass flux, heat source/sink, chemical reactivity, and Arrhenius activation energy. Nanoparticles exhibit thermophoretic and Brownian motion. The researchers numerically solved the equations and discussed the impacts of physical parameters on thermal, velocity, solutal, and heat transport. Rehman et al. [21] studied the effects of radiative heat on the bio-convective flow of magnetized Reiner-Philippoff nanoparticles towards a wedge. The analysis included factors such as Darcy-Forchheimer flow, swimming microorganisms, heat source/sink, Arrhenius activation energy, and nonlinear thermal radiation. The study used computational methods to solve the transformed ordinary differential equations. It highlighted key variables affecting thermal behavior, velocity, microorganisms, solutal properties, friction factor coefficient, heat transport, and motile density. Notably, heat transport increased with heat source/sink but decreased with Brownian motion. The bioconvection Lewies and Peclet numbers exhibited a reverse pattern in microorganism profiles.

### 1.3 Problem statement

According to the explanation of section 1.2, non-Newtonian fluids such as hyperbolic tangent fluid, MHD models, nanofluids, and hedonics such as wedges or porous geometries have wide applications in the industry in the presence of phenomena such as radiation and magnetic field. In the present study, investigating the magnetohydrodynamic flow of a hyperbolic tangent nanofluid from a permeable wedge by considering heat transfer can be very useful, especially when the governing equations of the problem are solved analytically. The originality and novelty of this study are in the analytical solution using the hybrid analytical and numerical method (HAN-method). The governing equations of this problem were a group of differential equations with partial derivatives consisting of continuity, momentum, energy, and concentration equations, and the main purpose of using these mentioned constitutive equations in the current study is to investigate the melting process concept which was one novelty of this article. Three dimensionless nonlinear differential equations of the third order with ordinary derivatives are obtained using suitable similarity transformations. In these extracted dimensionless ODEs, there are 11 dimensionless parameters. In the analysis of this study, the changes in the parameters that significantly affect the velocity field were analyzed, and the impact of these parameters on the skin friction coefficient, Nusselt number, and Sherwood number were also discussed. Graphs are used to illustrate the results, which are examined to quantitatively discuss the effects of various factors on parameters like fluid concentration, temperature, and velocity.

### 1.4 Why the HAN method?

In this research, that method has been used due to the brilliant history of the HAN semi-analytical method. The semi-analytical HAN method was proposed for the first time in analyzing a problem with stretching disks by Ahmadi Azar and Jalili [22]. The first paper [22] published with the HAN method was about fluid flow and heat and mass transfer for a micropolar and electrically conductive fluid in the presence of a magnetic field, after which this method was able to perform the coupled nonlinear ordinary differential equations well and analytically solve relatively more accurate than previous methods. In another study [23] similar to the previous article, the problem was two disks. However, with this difference, that study was the non-transient forced non-Newtonian MHD Reiner-Rivlin viscoelastic fluid motion that was constrained between two plates. The magnetic field was also present in this model. Ahmadi Azar and Jalili [24] used the HAN method in another problem that, unlike the equations used in the previous articles [22,23], this problem was in a porous medium with semi-infinite boundary conditions. The continuous flow of an incompressible Newtonian electrically conducting non-Darcy Casson fluid on a stretchy plate that is permeable vertically and incorporates a magnetic field is studied in this work. Using similarity transformations, the constitutive equations are converted from a system of nonlinear partial differential equations (PDEs) into a system of nonlinear ordinary differential equations (ODEs). Another study [25] applied the HAN method to a different set of semi-infinite boundary conditions, specifically the well-known Emden-Chandrasekhar equation for self-gravitating isothermal gas spheres in the theory of stellar structures. The analytical solution obtained in this research proved significantly more accurate than previous studies. The HAN method was used in other studies with different physics and geometries [[26], [27], [28], [29]], which was able to solve the governing equations with very good accuracy compared to other methods.

## 2 Methodology

This paper examines the incompressible, forced convection, two-dimensional time-independent MHD flow of the tangent hyperbolic nanofluid on a permeable wedge with melting heat transfer and heat radiation. The problem's geometry is as follows.

Here, $T_{\infty }$ , is the temperature of the nanofluid far away from the wedge, $C_{\infty }$ , is the concentration of the nanofluid far away from the wedge, $T_{m}$ , is the temperature of the molten material from the surface of the wedge, $T_{0}$ , is the temperature of the solid material from the surface of the wedge. The temperature of the molten material is higher than the temperature of the solid material, and the temperature of the nanofluid far away from the wedge $\left(T_{m}>T_{0}\right)$ and $\left(T_{m}>T_{\infty }\right)$. The nanofluid's velocity distribution outside of the boundary layer is defined below [9,30,31]:

(1) $$U\left(x\right)=ax^{m}\text{,}$$

here, U(x) , is the velocity boundary layer of the nanofluid, a is the positive real constant. The magnetic field distribution in the fluid flow is defined as follows [9,30]:

(2) $$B\left(x\right)=B_{0}\;x^{\left(\frac{m-1}{2}\right)}\text{,}$$

Where, (x) , is the function of the magnetic field, $B_{0}$ is the constant of the magnetic field perpendicular to the wedge walls. The function of permeability for the porous wedge is defined below [9,30]:

(3) $$K\left(x\right)=K_{0}\;x^{\left(1-m\right)}\text{,}$$

here, (x) , is the permeability function for the porous wedge, $K_{0}$ is the permeability constant for the porous wedge [9,30], which can be both positive and negative [32,33]. Normally, the amount of permeability is considered positive, but the negative amount of permeability can be seen in metamaterials. Metamaterials are artificial materials with extraordinary physical properties that are not available in natural materials. For instance, single-negative metamaterials have negative permeability or permittivity, while both are negative in double-negative metamaterials [32,33]. In Eqs. (1), (2), (3), the parameter of the m is defined as follows [9,30]:

(4) $$m=\frac{\beta_{1}}{2-\beta_{1}}\text{,}$$

in Eq. (4), m is the wedge angle parameter, and it is defined between 0 and 1 (0≤m≤1), and $\beta_{1}=\Omega /\pi$. Ω denotes the wedge's angle, and the Hartree pressure gradient is denoted by $\beta_{1}$. As the wedge angle parameter is 0, it means that the wedge walls are vertical, and when the wedge angle parameter is 1, the wedge walls are horizontal. The stress tensor for the hyperbolic tangent fluid equation is as follows [9,30,[34], [35], [36], [37], [38]]:

(5) $$\tau =\left(\left(\mu_{0}+\mu_{\infty }\right)\tanh\;{\left(\Gamma \dot{\Omega }\right)}^{n}+\mu_{\infty }\right)\dot{\Omega }\text{,}$$

in Eq. (5), the material constant of time is denoted by Γ, and the power law index is denoted by n, the null shear rate viscosity is denoted by $\mu_{0}$, the infinite shear rate viscosity is denoted by $\mu_{\infty }$, and for instance, when the power law is equal to 1, the fluid behaves like a Newtonian fluid.

Consider that $\mu_{\infty }=0$, and we are focusing on the hyperbolic tangent fluid, which represents shear thinning behavior when $\Gamma \dot{\Omega }<1$ and expression for τ reduces into:

(6) $$\tau =\mu_{0}\dot{\Omega }\tanh\;{\left(\Gamma \dot{\Omega }\right)}^{n}=\mu_{0}\dot{\Omega }\tanh\;{\left(\Gamma \dot{\Omega }-1+1\right)}^{n}=\mu_{0}\dot{\Omega }\left(-1+n\left(\Gamma \dot{\Omega }+1\right)\right)\text{,}$$

The parameter of $\dot{\Omega }$ in Eq. (5) is defined as follows [9,30,[34], [35], [36], [37], [38]]:

(7) $$\dot{\Omega }=\sqrt{\frac{1}{2}\;\sum_{m}\sum_{k}{\dot{\Omega }}_{mk}{\dot{\Omega }}_{km}}=\sqrt{\frac{1}{2}A}\text{,}$$

In Eq. (7), the second-order strain rate tensor invariant is denoted by A and considered as follows [9,30,[34], [35], [36], [37], [38]]:

(8) $$A=\frac{1}{2}tr{\left[{\left(\nabla V\right)}^{T}+\left(\nabla V\right)\right]}^{2}\text{,}$$

Here, in Eq. (8), the velocity vector is denoted by V [9,30,[34], [35], [36], [37], [38]]. The constitutive equations of the continuity, momentum, energy, and concentration for a two-dimensional steady-state non-compressible nanofluid flow considering heat and mass transfer are as follows [30]:

(9) $$\frac{\partial u}{\partial x}+\frac{\partial v}{\partial y}=0\text{,}$$

(10) $$u\frac{\partial u}{\partial x}+v\frac{\partial u}{\partial y}=U\left(x\right)\frac{\partial U\left(x\right)}{\partial x}+\nu \left(\left(1-n\right)+\sqrt{2}n\Gamma \left(\frac{\partial u}{\partial y}\right)\right)\frac{\partial^{2}u}{\partial y^{2}}+\left(\frac{\sigma {\left(B\left(x\right)\right)}^{2}}{\rho_{f}}+\frac{\nu }{K\left(x\right)}\right)\left(U\left(x\right)-u\right)\text{,}$$

(11) $$u\frac{\partial T}{\partial x}+v\frac{\partial T}{\partial y}=\alpha \frac{\partial^{2}T}{\partial y^{2}}+\frac{\kappa_{T}D_{B}}{C_{s}C_{p}}\frac{\partial^{2}C}{\partial y^{2}}-\frac{1}{\rho C_{p}}\frac{\partial q_{r}}{\partial y}+\Lambda \left(D_{B}\frac{\partial C}{\partial y}\frac{\partial T}{\partial y}+\frac{D_{T}}{T_{\infty }}{\left(\frac{\partial T}{\partial y}\right)}^{2}\right)\text{,}$$

(12) $$u\frac{\partial C}{\partial x}+v\frac{\partial C}{\partial y}=\frac{\kappa_{T}D_{B}}{T_{m}}\frac{\partial^{2}T}{\partial y^{2}}+\frac{D_{T}}{T_{\infty }}\frac{\partial^{2}T}{\partial y^{2}}+D_{B}\frac{\partial^{2}C}{\partial y^{2}}\text{,}$$

**Fig. 1:**
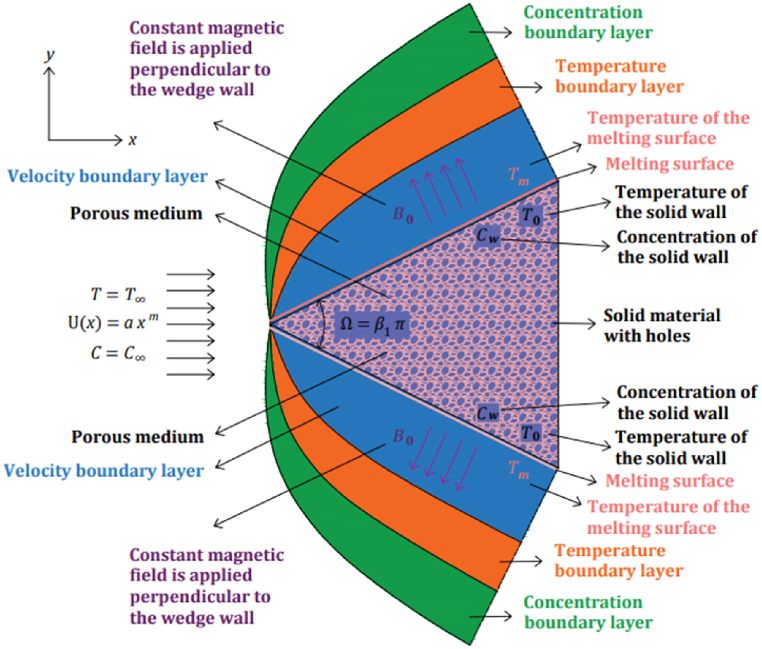
The geometry of the fluid flow and heat transfer on a permeable wedge.

Where in Eqs. (9), (10), (11), (12), u(x,y) , is the velocity along x-axis and it is denoted by u, v(x,y) , is the velocity along y-axis and it is denoted by v, T(x,y) is denoted by T, and C(x,y) , is denoted by C. The concentration in dimensional form is shown by C, the temperature in dimensional form is shown by T, the thermophoresis diffusion is demonstrated with $D_{T}$, the Brownian diffusion is demonstrated with $D_{B}$, $\Lambda ={\left(\rho c\right)}_{p}/{\left(\rho c\right)}_{f}$ , states the coefficient of heat capacity of nanofluid to normal fluid, the viscosity of nanofluid is denoted with ν, the thermal diffusivity of nanofluid is showed with $\alpha =k/{\left(\rho c\right)}_{f}$, the power law index of the fluid is denoted with n, the electrical conductivity is denoted with σ, and the ratio of thermal diffusion is demonstrated by $\kappa_{T}$. The proper boundary conditions for PDEs of (9–12) and the geometry of the problem (shown in Fig. 1) are as follows [9,30]:

(13) $$u=0,T=T_{m},v=0,k\frac{\partial T}{\partial y}=\rho v\left(C_{s}\left(T_{m}-T_{0}\right)+\lambda \right),C=C_{w},\text{at}\;y=0$$

(14) $$u=U\left(x\right),v=0,T=T_{\infty },C=C_{\infty }\text{,}$$

Here, in Eqs. (13), (14), the latent heat of the nanofluid is demonstrated via λ, the thermal conductivity of the nanofluid is displayed by k, and the concentration of the nanofluid at the surface of the wedge is displayed with $C_{w}$, the concentration of the nanofluid far away from the solid surface of the wedge is shown with $C_{\infty }$, the heat capacity of the solid surface is shown with $C_{s}$, and the solid's density is presented through ρ. The radiation heat flux can be expressed using the Rosseland approximation and written as [9,30]:

(15) $$q_{r}=-\frac{4\sigma^{*}}{\kappa^{*}}\frac{\partial T^{4}}{\partial y}\text{,}$$

In Eq. (15), the Stefan-Boltzmann constant is shown with the $\sigma^{*}$, and the absorption constant is presented with $\kappa^{*}$. By the use of the Taylor series expansion of $T^{4}$ about the temperature of $T_{\infty }$, the following equation is constructed as below [9,30]:

(16) $$T^{4}≅-3T_{\infty }^{4}+4T_{\infty }^{3}T\text{,}$$

According to Eq. (16), by substituting the $T^{4}$, in Eq. (15), The radiation heat flux will be changed as follows [9,30]:

(17) $$q_{r}=-\frac{4\sigma^{*}}{\kappa^{*}}\frac{\partial \left(4T_{\infty }^{3}T-3T_{\infty }^{4}\right)}{\partial y}\text{,}$$

So, by substituting Eq. (17) in the energy equation of Eq. (11) will be changed to the following form:

(18) $$u\frac{\partial T}{\partial x}+v\frac{\partial T}{\partial y}=\left(\alpha +\frac{16\sigma^{*}T_{\infty }^{2}}{3\rho C_{p}\kappa^{*}}\right)\frac{\partial^{2}T}{\partial y^{2}}-\frac{1}{\rho C_{p}}\frac{\partial q_{r}}{\partial y}+\Lambda \left(D_{B}\frac{\partial C}{\partial y}\frac{\partial T}{\partial y}+\frac{D_{T}}{T_{\infty }}{\left(\frac{\partial T}{\partial y}\right)}^{2}\right)\text{,}$$

The stream function of ψ(x,y) , is a function dependent on the changes of x and y, which is represented by ψ and is defined in Eq. (19) as follows:

(19) $$u=\frac{\partial \psi }{\partial y},v=-\frac{\partial \psi }{\partial x}\text{,}$$

Since Eqs. (9), (10), (11), (12) are difficult to solve due to their multivariate nature, the dimensionless similarity variables can be used to reduce the independent variables. These similarity variables are as follows [9,30]:

(20) $$\xi ={y\left(\frac{\left(m+1\right)U\left(x\right)}{2x\nu }\right)}^{1/2},\psi =f\left(\xi \right){\left(\frac{2x\nu U\left(x\right)}{m+1}\right)}^{1/2},\theta \left(\xi \right)=\frac{T-T_{\infty }}{T_{m}-T_{\infty }},\phi \left(\xi \right)=\frac{C-C_{\infty }}{C_{m}-C_{\infty }}\text{,}$$

Where ξ is the dimensionless independent variable, $f^{\prime }\left(\xi \right)$ *,* is the dimensionless velocity quantity, θ(ξ) , is the dimensionless temperature quantity, ϕ(ξ) , is the dimensionless concentration quantity. By substituting Eq. (20) in Eqs. (10), (12), (18), the following third-order nonlinear coupled system of ODEs will be concluded:

(21) $$\left(1-n+nWi\;\left(\frac{d^{2}f\left(\xi \right)}{d\xi^{2}}\right)\right)\frac{d^{3}f\left(\xi \right)}{d\xi^{3}}+f\left(\xi \right)\frac{d^{2}f\left(\xi \right)}{d\xi^{2}}+\left(\frac{1}{K_{p}}+M\right)\left(1-\frac{df\left(\xi \right)}{d\xi }\right)+\left(\frac{2m}{m+1}\right)\left(1-{\left(\frac{df\left(\xi \right)}{d\xi }\right)}^{2}\right)=0\text{,}$$

(22) $$\left(1+\frac{4Rd}{3}\right)\frac{d^{2}\theta \left(\xi \right)}{d\xi^{2}}+\mathrm{Pr}\left(D_{4}\frac{d^{2}\phi \left(\xi \right)}{d\xi^{2}}+Nb\frac{d\theta \left(\xi \right)}{d\xi }\frac{d\phi \left(\xi \right)}{d\xi }+Nt{\left(\frac{d\theta \left(\xi \right)}{d\xi }\right)}^{2}+f\left(\xi \right)\frac{d\theta \left(\xi \right)}{d\xi }\right)=0\text{,}$$

(23) $$\frac{d^{2}\phi \left(\xi \right)}{d\xi^{2}}+PrLef\left(\xi \right)\frac{d\phi \left(\xi \right)}{d\xi }+\frac{d^{2}\theta \left(\xi \right)}{d\xi^{2}}\left(PrLeSr+\frac{Nt}{Nb}\right)=0\text{.}$$

Here, $Wi={\left(\left(\Gamma^{2}\left(m+1\right){\left(U\left(x\right)\right)}^{3}\right)/\left(\nu x\right)\right)}^{1/2}$ is the Weissenberg number, $K_{p}=\left(aK_{0}\left(m+1\right)\right)/\left(2\nu \right)$ is the permeability of the medium, $M=\left(2\sigma B_{0}^{2}\right)/\left(\rho_{f}a\left(m+1\right)\right)$ is the parameter of the magnetic field, $Rd=\left(4\sigma^{*}T_{\infty }^{3}\right)/\left(\kappa^{*}\kappa \right)$ , is the parameter of thermal radiation, Pr=ν/α is the Prandtl number, $D_{4}=\left(\kappa_{T}D_{B}\left(C_{w}-C_{\infty }\right)\right)/\left(C_{s}C_{p}\left(T_{m}-T_{\infty }\right)\right)$ , is the Dufour number, $Nb=\left(\Lambda D_{B}\left(C_{w}-C_{\infty }\right)\right)/\nu$ is the parameter of Brownian diffusion, $Nt=\left({\Lambda D}_{T}\left(T_{m}-T_{\infty }\right)\right)/\left(\nu T_{\infty }\right)$ , is the thermophoresis parameter, $Le=\alpha /D_{B}$ , is the Lewis number, and $Sr=\left(\kappa_{T}D_{B}\left(T_{m}-T_{\infty }\right)\right)/\left(T_{m}\nu \left(C_{w}-C_{\infty }\right)\right)$ , is the Soret number. The similarity transformation of Eq. (20) will affect the boundary conditions of Eqs. (13), (14) and reduced the mentioned boundary conditions into the following form:

(24) $$\frac{df\left(\xi \right)}{d\xi }=0,\frac{d\theta \left(\xi \right)}{d\xi }=1,B\frac{d\theta \left(\xi \right)}{d\xi }+Prf\left(\xi \right)=0,\phi \left(\xi \right)=1,\text{when}\;\xi =0\text{,}$$

(25) $$\frac{df\left(\xi \right)}{d\xi }=1,\theta \left(\xi \right)=0,\phi \left(\xi \right)=0,\text{when}\;\xi =\infty \text{.}$$

Here, the effect of melting in the mathematical form is shown with $B=\left(C_{f}\left(T_{m}-T_{0}\right)\right)/\left(\lambda +C_{s}\left(T_{m}-T_{0}\right)\right)$. Eqs. (24), (25) are the boundary conditions of Eq. (21), (22), (23). Likewise, the dimensionless skin friction coefficient ($C_{f}$), Nusselt number (Nu), and Sherwood number (Sh) can be calculated as follows [9,30,[34], [35], [36], [37], [38]]:

(26) $$C_{f}=\frac{\left(1-n\right)}{\sqrt{{Re}_{x}}}\left(\frac{d^{2}f\left(0\right)}{d\xi^{2}}\right)+\left(\frac{n}{2}\right)Wi{\left(\frac{d^{2}f\left(0\right)}{d\xi^{2}}\right)}^{2}\text{,}$$

(27) $$Nu=-\left(\left(\sqrt{{Re}_{x}}\right)\left(1+\left(\frac{4}{3}\right)Rd\right)\right)\left(\frac{d\theta \left(0\right)}{d\xi }\;\right)\text{,}$$

(28) $$Sh=-\left(\sqrt{{Re}_{x}}\right)\left(\frac{d\phi \left(0\right)}{d\xi }\;\right)\;\text{.}$$

Here, in Eqs. (26), (27), (28), ${Re}_{x}$ , is the local Reynolds number [30].

## 3 Mathematical scheme

The HAN method has been used in various studies [39,40] for resolving nonlinear ordinary differential equations, and its methodology has been presented in detail. To utilize the HAN technique for obtaining the semi-analytical answer of the set of ordinary differential ODEs (21–23), we initially presume that the three polynomials with constant coefficients act as analytical solutions when Wi=0.5, B=0.0, n=0.5, M=0.1, $K_{p}=-5$, Pr=3.0, Le=0.5, $D_{4}=0.1$, Sr=0.1, Rd=2, Nb=0.2, Nt=0.2, and m=0.5 are as follows:

(29) $$f\left(\xi \right)=\sum_{i=0}^{9}a_{i}\xi^{i},\theta \left(\xi \right)=\sum_{i=0}^{9}c_{i}\xi^{i},\phi \left(\xi \right)=\sum_{i=0}^{9}d_{i}\xi^{i}$$

Eq. (29) shows that there are 30 unknown coefficients that can be determined by creating 30 algebraic equations. However, the boundary conditions in Eqs. (24), (25) alone are insufficient for creating all 30 equations. Instead of using the boundary conditions of the problem for making algebraic equations (in which they are always insufficient), the algebraic can be constructed using the numerical solution of ODEs (21–23) via a numerical solution. Deciding which numerical method with the help of which mathematical software is highly dependent on the form of the differential equations. Table (1) demonstrates the numerical solution by the Runge-Kutta method.

**Table 1:** The numerical solution to the problem using the Runge-Kutta method via Maple software.

ξ	f(ξ)	$f^{\prime }\left(\xi \right)$	θ(ξ)	$\theta^{\prime }\left(\xi \right)$	ϕ(ξ)	$\phi^{\prime }\left(\xi \right)$
0	0	0	1.00000000000000	−0.435779284828820	1.00000000000000	−0.563213396341648
1	0.467993936563998	0.806980443838309	0.539096930632243	−0.456316797640900	0.497619372689582	−0.422604193485592
2	1.39904284284803	0.990348218869182	0.171167899626798	−0.251880347656307	0.171134108815754	−0.230175863724838
3	2.39676008863283	0.999910761077042	0.0268159751590013	−0.0619114701503303	0.0296364003812728	−0.0666539043157664
4	3.39674495078888	1.00000000000000	0	−0.0070853395449190	0	−0.00805745948855878

Since numerical solutions are not exact, we can treat them as approximate boundary conditions. The problem's boundary conditions may only provide ten algebraic equations, which is not enough. However, we can obtain all the required algebraic equations by using the approximate boundary conditions listed in Table (1). By constructing 30 algebraic equations, we can determine the 30 unknown coefficients of Eq. (29), which will give us the semi-analytical solutions of ODEs (21–23) as follows:

(30) $$f\left(\xi \right)=-0.00004179315773\;\xi^{9}+0.0007272563133\;\xi^{8}-\;0.004902079642\;\xi^{7}+0.01505631191\;\xi^{6}-\;0.01626670310\;\xi^{5}+0.001237224635\;\xi^{4}-\;0.1224676045\;\xi^{3}+0.5946513241\;\xi^{2}\text{,}$$

(31) $$\theta \left(\xi \right)=0.00004389361336\;\xi^{9}-\;0.0008592172362\;\xi^{8}+0.006764579442\;\xi^{7}-\;0.02644448162\;\xi^{6}+0.04951495678\;\xi^{5}-\;0.03324610037\;\xi^{4}+0.02446046405\;\xi^{3}-\;0.04535787920\;\xi^{2}-\;0.4357792848\;\xi +1\text{,}$$

(32) $$\phi \left(\xi \right)=-0.00006860923586\;\xi^{9}+0.001250503581\;\xi^{8}-\;0.009342331334\;\xi^{7}+0.03677523251\;\xi^{6}-\;0.08132566587\;\xi^{5}+0.09524391635\;\xi^{4}-\;0.03497920048\;\xi^{3}+0.05327892352\;\xi^{2}-\;0.5632133963+1\text{,}$$

**Fig. 2:**
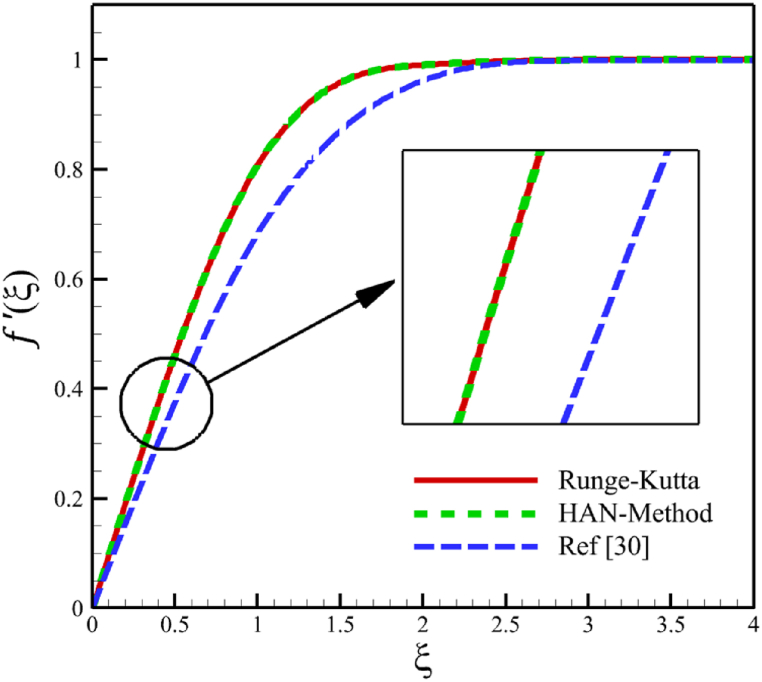

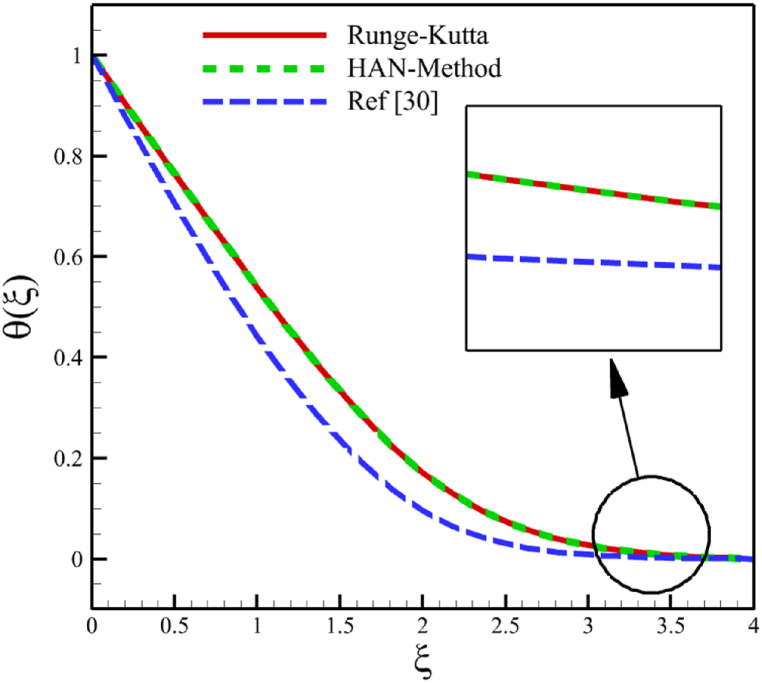

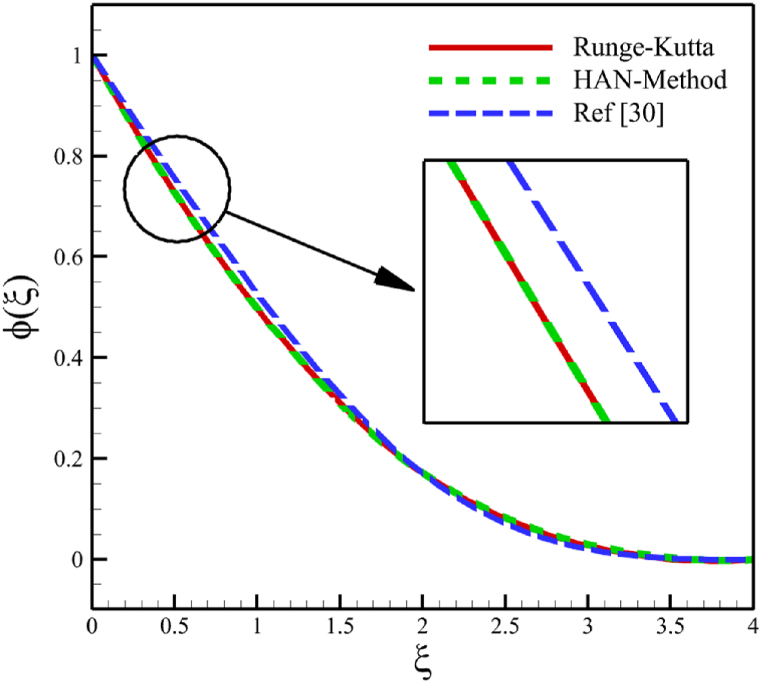

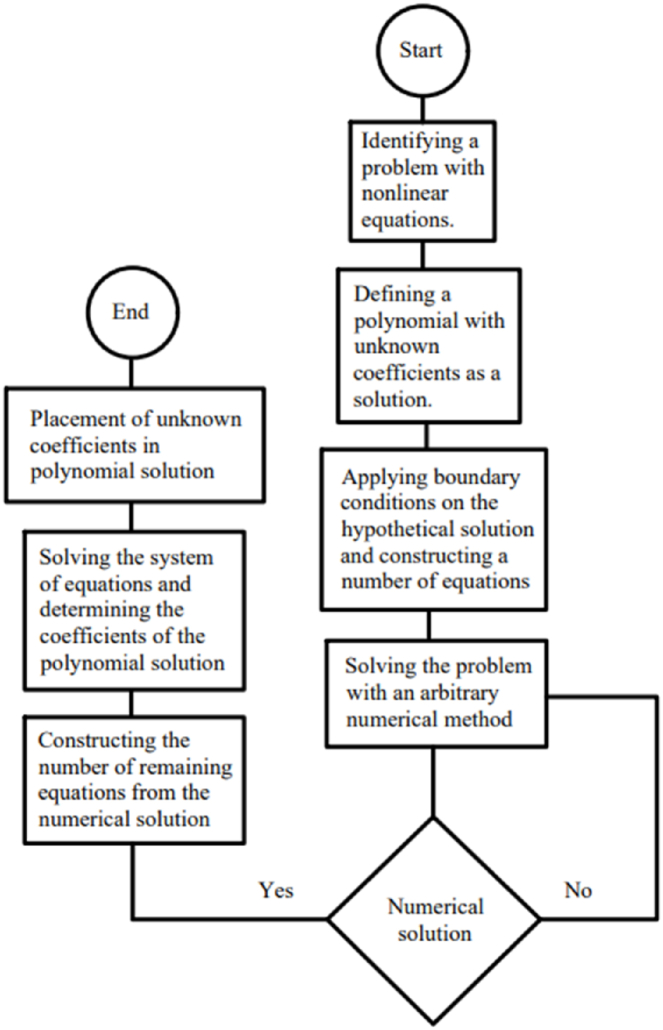
a - The comparison of velocity results.**b -** The comparison of temperature results.**c -** The comparison of concentration results.**d -** The flowchart of the HAN method.

Here, Eqs. (30), (31), (32) are the HAN solutions. A comparison of the analytical solutions (30–32) with reference [30] can be found in Figs. (2a-2c) to validate current results. Summarizing how the HAN method works is demonstrated through a flowchart illustrated in Fig. 2d.

Regarding the HAN method, it is worth noting that, firstly. At the same time, we have primarily utilized it to convert numerical solutions of ODEs to analytical solutions, and it can also be applied to PDEs. Secondly, this method is not restricted to any particular numerical technique, and lastly, any software capable of numerically solving differential and algebraic equations can be effectively utilized with this method.

## 4 Results and discussion

### 4.1 Overview of the results

In this part, by using Eqs. (21), (22), (23), the effect of parameter changes on important quantities such as velocity, concentration, temperature, Nusselt number, skin friction coefficient, and Sherwood number are quantitatively presented, and the corresponding graphs are also displayed. This subsection explains the mechanism by which the article's results were obtained. In Eqs. (21), (22), (23), there are a number of parameters, and changing each of these parameters alone, while the other parameters do not change, affects the parameters in the equations. The results in this article have been extracted in such a way that certain parameters of this problem, including Weissenberg number, power law index, the permeability of the medium, magnetic field parameter, wedge angle parameter, melting heat transfer, and local Reynolds number, were selected, and the effect of changing each of these parameters on parameters such as velocity, concentration, temperature, Nusselt number, skin friction coefficient, and Sherwood number was investigated.

### 4.2 The effect of the Weissenberg number

**Fig. 3:**
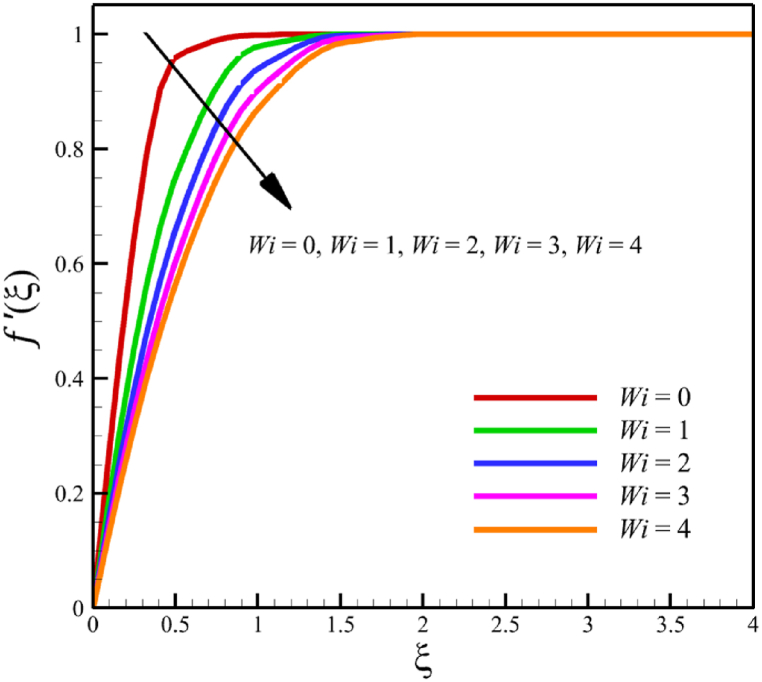

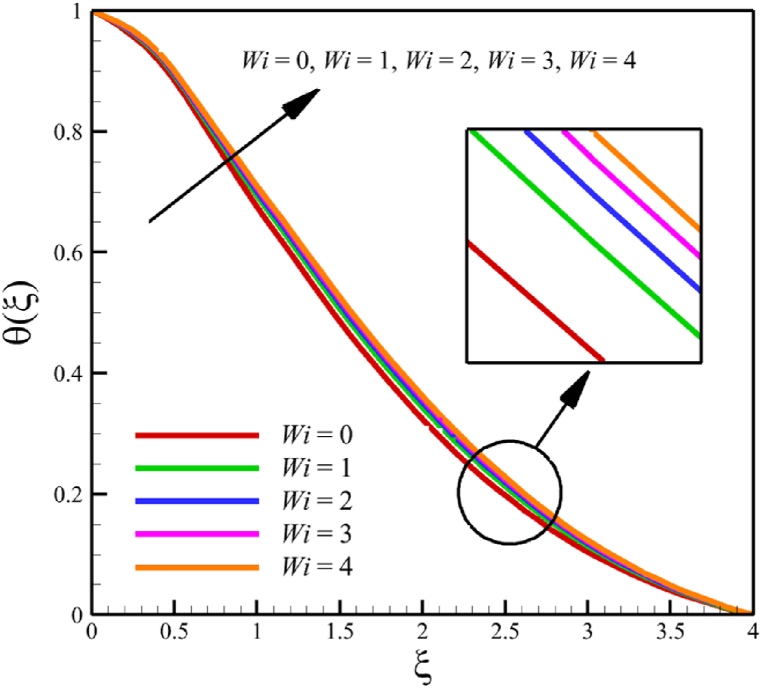

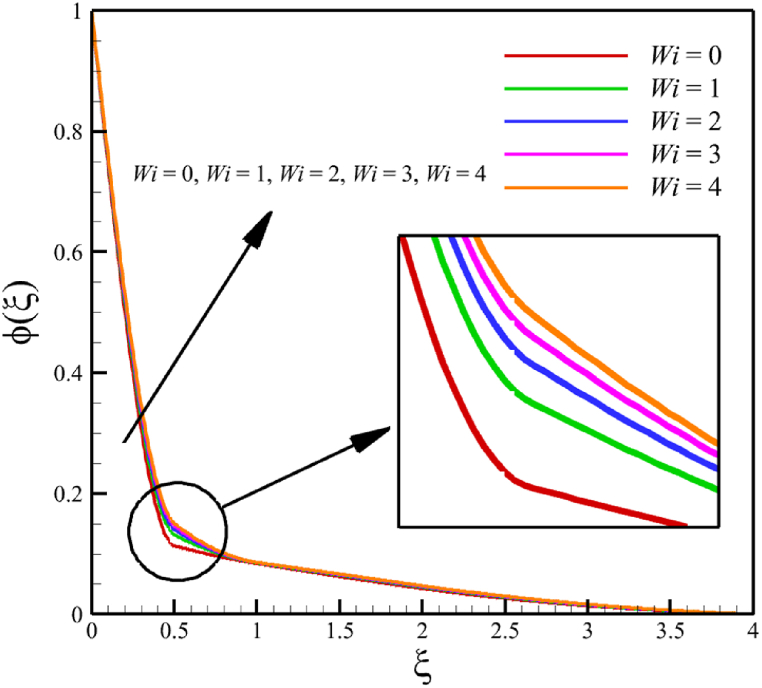

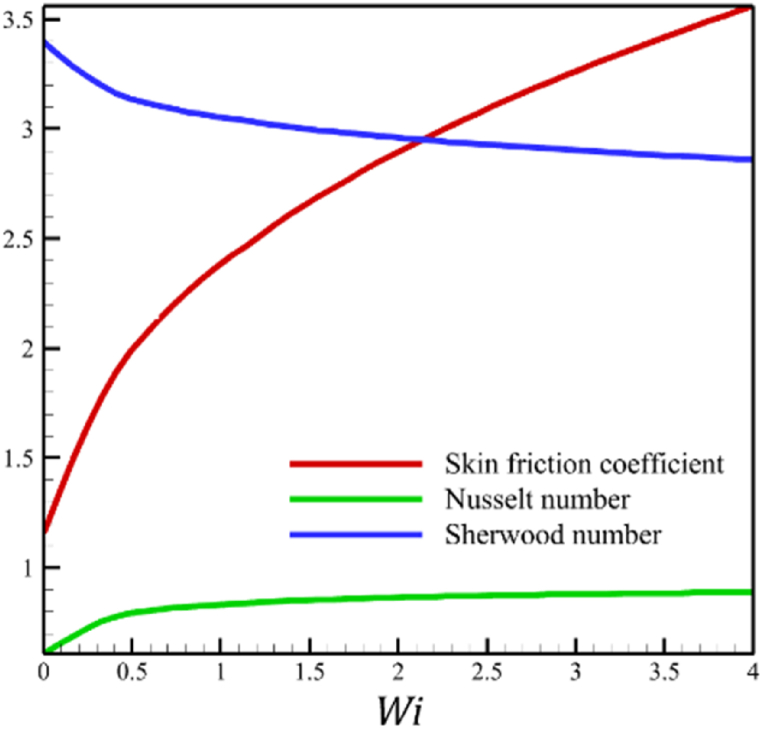
a - The effect on the velocity quantity when the parameter Weissenberg number changes from 0 to 4.**b -** The effect on the temperature quantity when the parameter Weissenberg number changes from 0 to 4.**c -** The effect on the concentration quantity when the parameter Weissenberg number changes from 0 to 4.**d -** The effect on the skin friction coefficient, Sherwood number, and Nusselt number when the parameter Weissenberg number changes from 0 to 4.

The effect of changes in the Weissenberg number in the current sub-section is investigated. Since the Weissenberg number has already been defined in Section 2 (Methodology), it has a direct relationship with values such as the velocity distribution of the nanofluid outside the boundary layer, the time material constant, and the wedge angle parameter, and it has an inverse relationship with the viscosity of the nanofluid. By increasing the Weissenberg number from 0 to 4, it can be concluded that one of the mentioned parameters, including the velocity of the nanofluid outside the boundary layer, or the material time constant or the wedge angle parameter, has increased. However, from the increase in Weissenberg's number, it can be concluded that the viscosity of a nanofluid is decreasing. It was said that the Weissenberg number can be changed by changing the wedge angle parameter, but it should be considered that the value of the wedge angle parameter must be between 0 and 1. Therefore, according to Fig. 3a, by increasing the Weissenberg number from 0 to 4, while other parameters remained B=2.5, n=0.8, M=5.0, $K_{p}=5.0$, Pr=4.0, Le=3, $D_{4}=0.4$, Sr=0.4, Rd=10, Nb=0.4, Nt=0.4, and m=0.8, it causes a drop in the velocity of the fluid. So, the average velocity of the fluid changes from 0.884361695 to 0.824199582, which causes a 6.802885442 % reduction in the fluid's velocity. According to Fig. 3b, increasing the Weissenberg number from 0 to 4 causes a growth in the temperature of the fluid. So, the average temperature of the fluid changes from 0.412486756 to 0.433296061, which causes a 5.044841973 % growth in the fluid's temperature. According to Fig. 3c, increasing the Weissenberg number from 0 to 4 causes a growth in the concentration of the fluid. So, the average concentration of the fluid changes from 0.149748922 to 0.15593566, which causes a 4.131407370 % growth in the fluid's concentration. According to Fig. 3d, by increasing the Weissenberg number from 0 to 4, the skin friction coefficient and the Nusselt number were increased while the Sherwood number decreased. As the Weissenberg number changed from 0 to 4, the skin friction coefficient changed from 1.158986491 to 3.561032955, which shows 207.2540519 % growth in this coefficient, and the Nusselt number changes from 0.606639888 to 0.888110204, which shows 46.39825398 % growth in this number. Unlike the skin friction coefficient and the Nusselt number, the Sherwood number will decrease from 3.395327299 to 2.860273227, which shows a 15.75854181 % drop in this number. The Weissenberg number is more effective on the skin friction coefficient than the Nusselt or Sherwood number because the Weissenberg number exists in Eq. (26); the skin friction coefficient is a function of the Weissenberg number.

### 4.3 The effect of the power law index

**Fig. 4:**
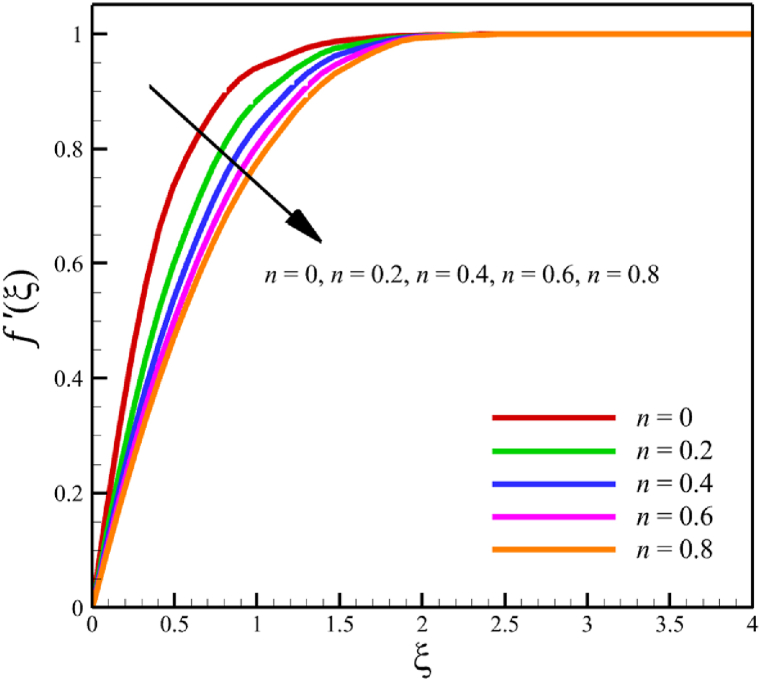

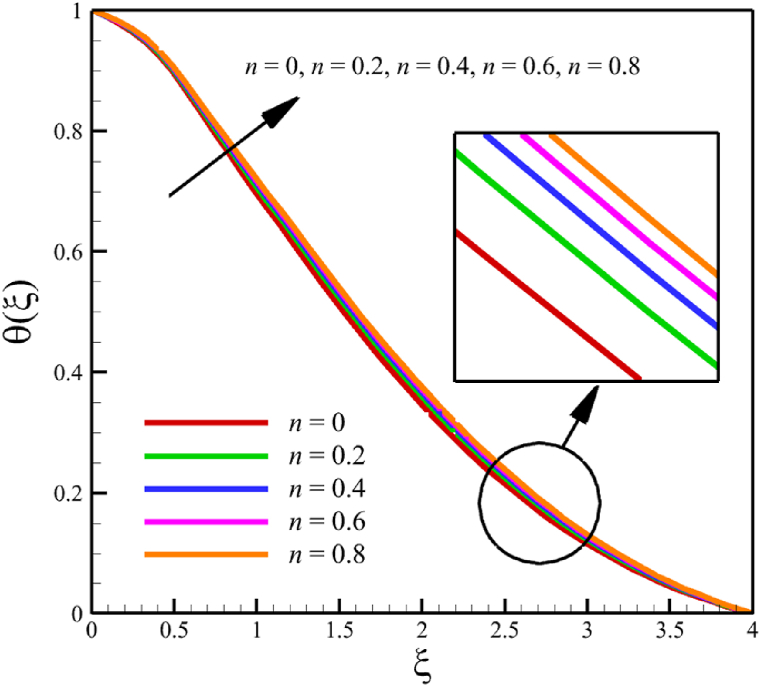

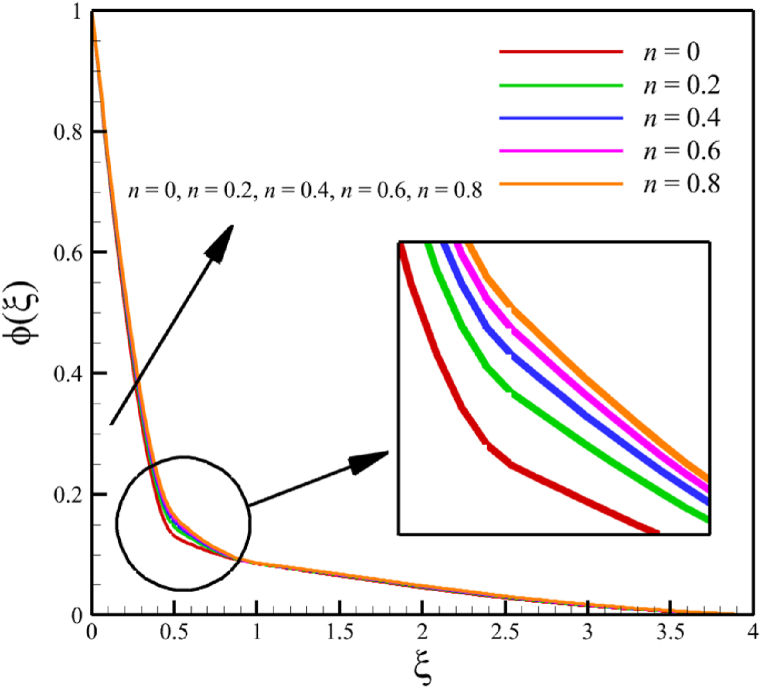

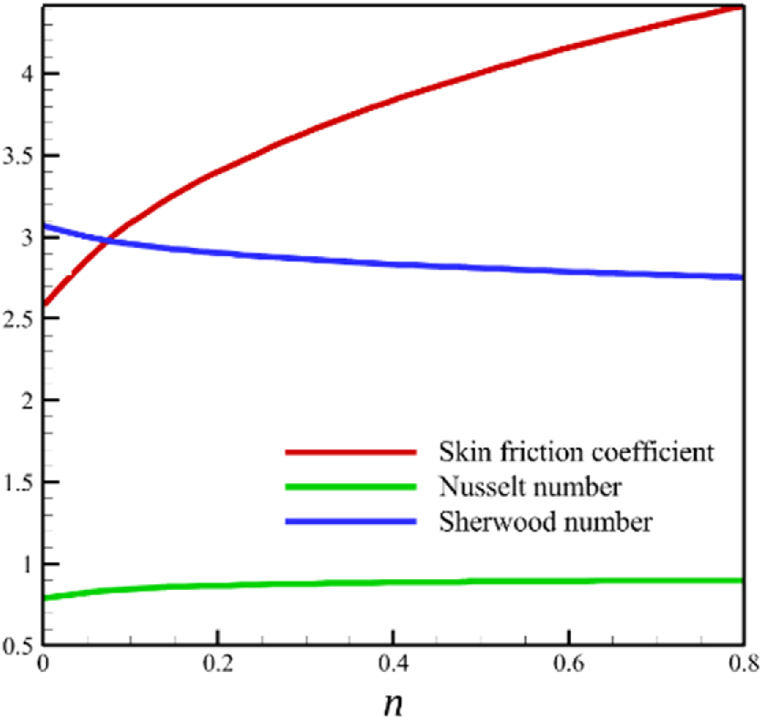
a - The effect on the velocity quantity when the power law index changes from 0 to 0.8.**b -** The effect on the temperature quantity when the power law index changes from 0 to 0.8.**c -** The effect on the concentration quantity when the power law index changes from 0 to 0.8.**d -** The effect on the skin friction coefficient, Nusselt number, and Sherwood number when the power law index changes from 0 to 0.8.

The effect of changes in the power law index is investigated in the current sub-section. Since the power law index has already been defined in Section 2 (Methodology), it can affect the fluid's velocity because it was appeared first in Eq. (5) and related to the stress tensor for hyperbolic tangent fluid. So, according to Eqs. (5), (26), the power law index directly affects both the fluid's velocity and the skin friction coefficient. Different power law indexes affect the stress tensor of the nanofluid, according to Figs. (4a-4d), the effects of changing the power law index on six dimensionless quantities are investigated. The power law index changes from 0 to 0.8 in Figs. (4a-4d) while other parameters are remained Wi=8.0, B=2.5, M=5.0, $K_{p}=5.0$, Pr=4.0, Le=3, $D_{4}=0.4$, Sr=0.4, Rd=10, Nb=0.4, Nt=0.4, and m=0.8. According to Fig. 4a, as the n parameter changes from 0 to 0.8, the average velocity of the fluid changes from 0.852034247 to 0.797917993, which is a 6.351417703 % drop in fluid's velocity. According to Fig. 4b, the average temperature changes from 0.424383887 to 0.440239156, a 3.736067623 % growth in fluid temperature. According to Fig. 4c, the average concentration changes from 0.152784259 to 0.15801291, a 3.422244565 % growth in fluid concentration. According to Fig. 4d, by increasing the power law index from 0 to 0.8, the skin friction coefficient and the Nusselt number were increased while the Sherwood number decreased. As the power law index changed from 0 to 0.8, the skin friction coefficient changes from 2.57871557 to 4.414905282, which shows 71.20559295 % growth in this coefficient, and the Nusselt number changes from 0.79198107 to 0.901576488, which shows 13.83813606 % growth in this number. Unlike the skin friction coefficient and the Nusselt number, the Sherwood number will decrease from 3.067370802 to 2.755126772, which shows a 10.17953323 % drop in this number. The power law index is more effective on the skin friction coefficient than the Nusselt or Sherwood number because the power law index parameter exists in Eq. (26); the skin friction coefficient is a function of the power law index parameter.

### 4.4 The effect of the permeability of the medium

**Fig. 5:**
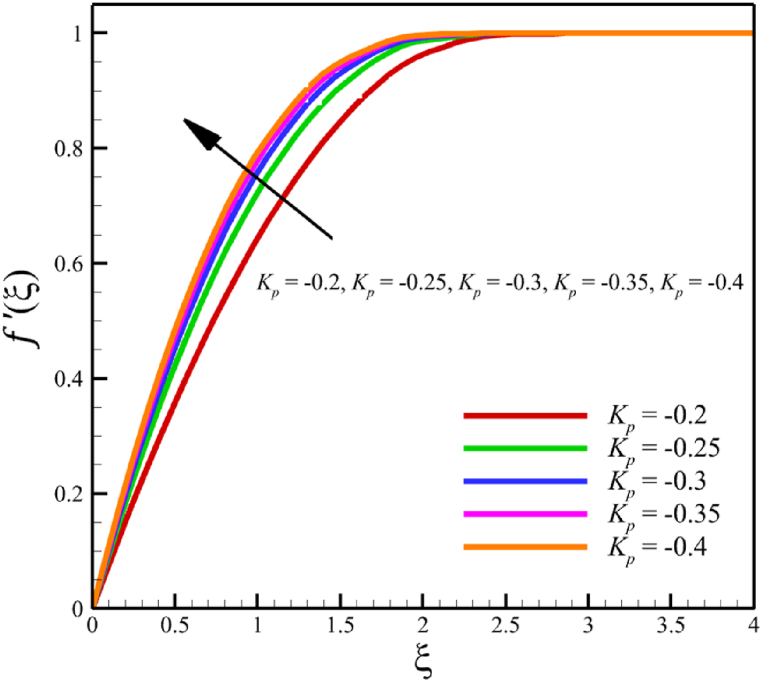

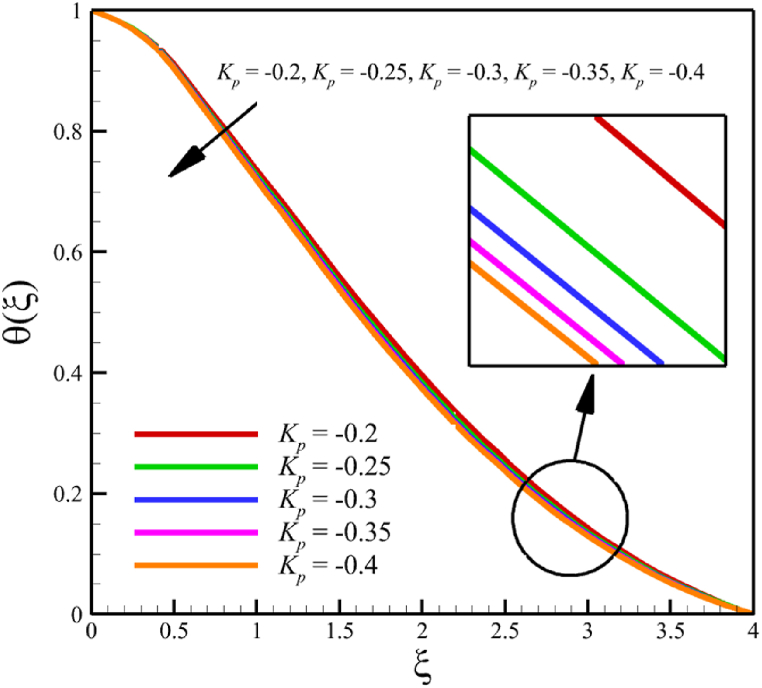

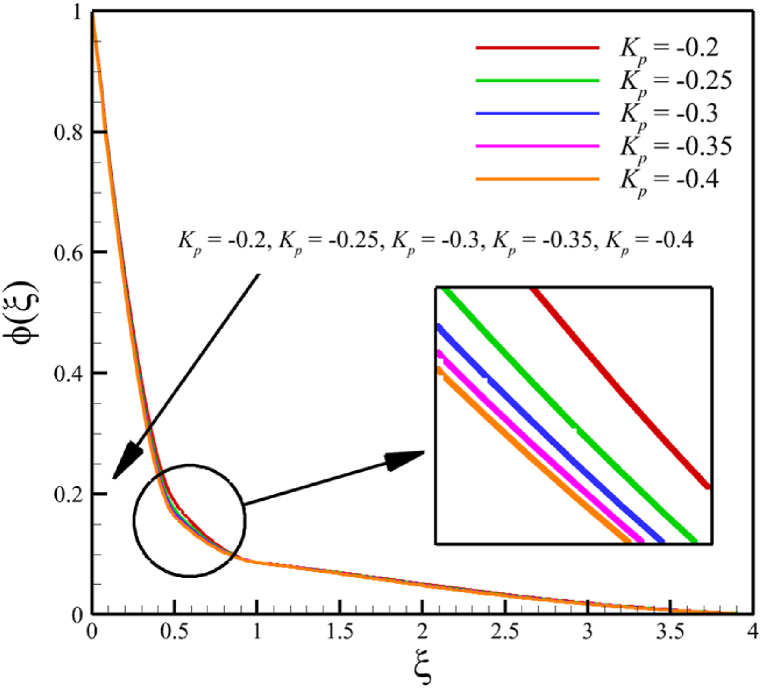

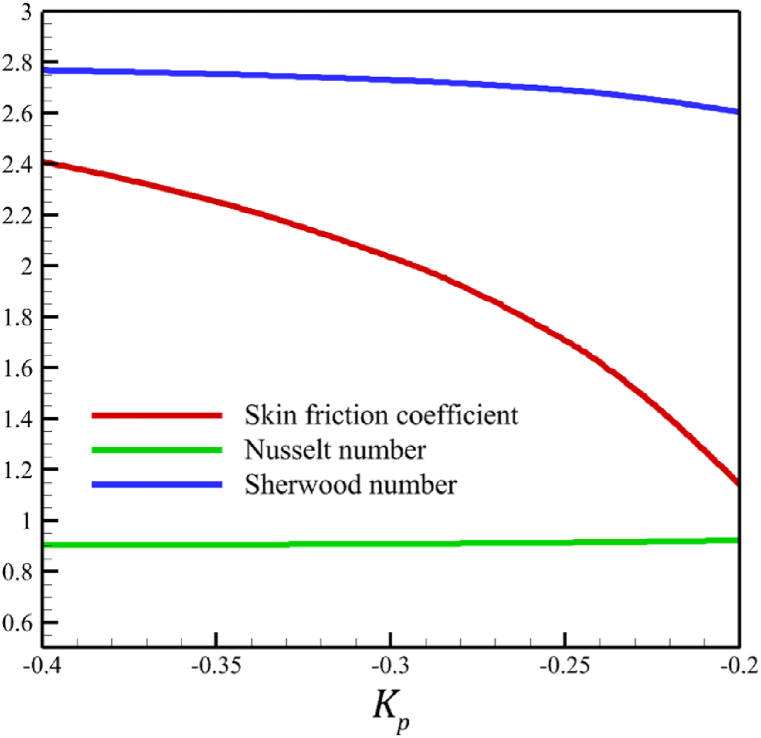
a - The effect on the velocity quantity when the negative permeability of the medium changes from −0.2 to −0.4.**b -** The effect on the temperature quantity when the negative permeability of the medium changes from −0.2 to −0.4.**c -** The effect on the concentration quantity when the negative permeability of the medium changes from −0.2 to −0.4.**d -** The effect on the Nusselt number, skin friction coefficient, and Sherwood number when the negative permeability of the medium changes from −0.2 to −0.4.

**Fig. 6:**
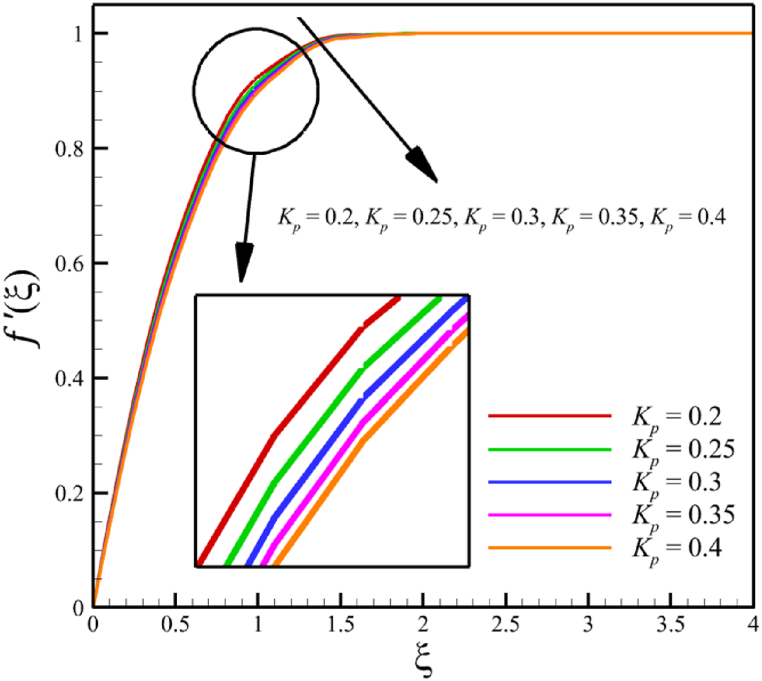

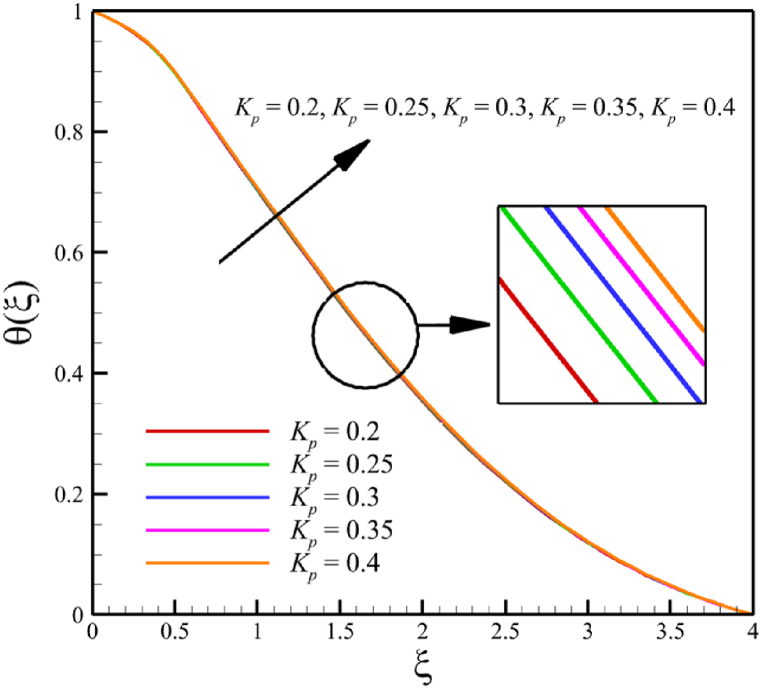

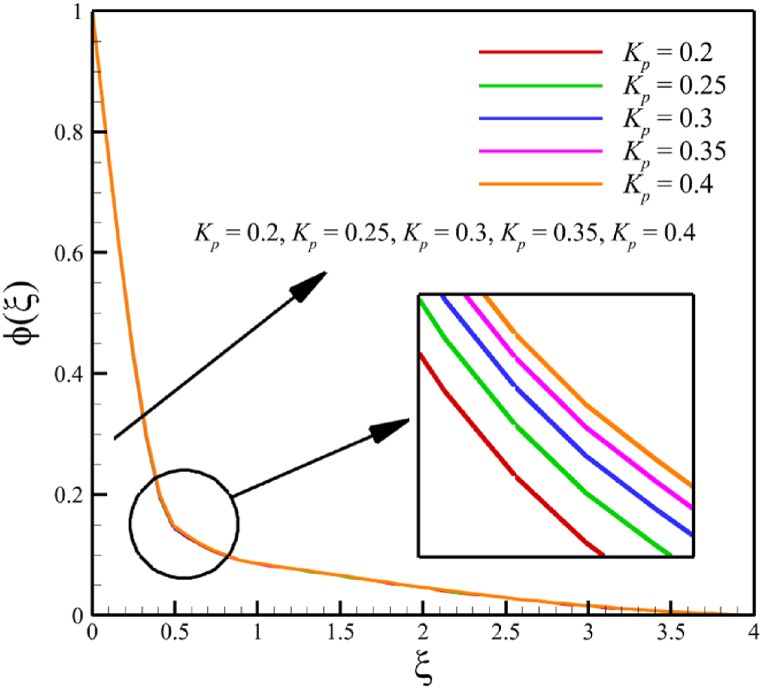

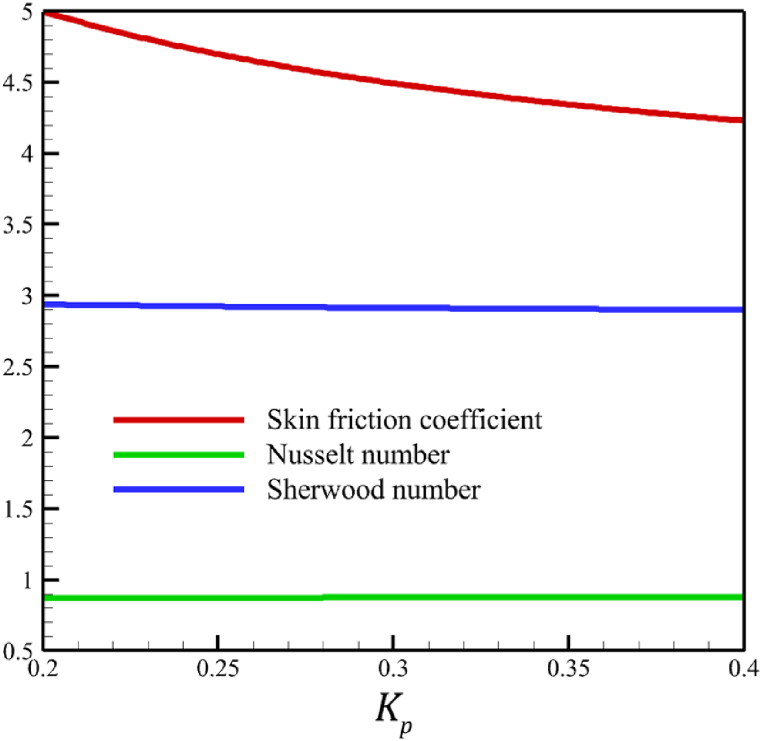
a - The effect on the velocity quantity when the positive permeability of the medium changes from 0.2 to 0.4.**b -** The effect on the temperature quantity when the positive permeability of the medium changes from 0.2 to 0.4.**c -** The effect on the concentration quantity when the positive permeability of the medium changes from 0.2 to 0.4.**d -** The effect on the skin friction coefficient, Nusselt number, and Sherwood number when the positive permeability of the medium changes from 0.2 to 0.4.

This subsection investigates the effect of both positive and negative permeability changes on the wedge. Since the permeability of the medium has already been defined in Section 2 (Methodology), it has a direct relationship with values such as the positive real constant (a), the constant of permeability for the porous wedge ($K_{0}$), and the wedge angle parameter (m) while it has an inverse relationship with the viscosity of the nanofluid. By increasing the medium's permeability, it can be concluded that one of the mentioned parameters, including the permeability constant for the porous wedge or the wedge angle parameter, has increased. However, just like the Weissenberg number, from the increase of the permeability of the medium, it can be concluded that the viscosity of a nanofluid is decreasing. Just like the Weissenberg number, considering that it was said that the medium's permeability can be changed by changing the wedge angle parameter, it should be considered that the value of the wedge angle parameter must be between 0 and 1. According to Eq. (21), because the medium's permeability is in the denominator, choosing values greater than one will decrease the effect of this parameter. Therefore, according to Figs. (5a-5d), the velocity, concentration profiles, temperature, skin friction coefficient, Sherwood number, and Nusselt number are affected by changing the permeability of the medium from −0.2 to −0.4, while other parameters have remained Wi=4.0, B=2.5, n=0.8, M=5.0, Pr=4.0, Le=3, $D_{4}=0.4$, Sr=0.4, Rd=10, Nb=0.4, Nt=0.4, and m=0.8. Among these effects, the velocity profile is more affected than the rest of the quantities. According to Fig. 5a, as the permeability of the medium changes from −0.2 to −0.4, the average velocity increases from 0.756695872 to 0.802749855, a 6.086194560 % growth in the fluid's velocity. According to Fig. 5b, as the permeability of the medium changes from −0.2 to −0.4, the average temperature decreases from 0.450689928 to 0.439071781, which is a 2.577858141 % drop in the fluid's temperature. According to Fig. 5c, as the permeability of the medium changes from −0.2 to −0.4, the average concentration decreases from 0.161477708 to 0.157727143, which is a 2.322651867 % drop in fluid concentration. According to Fig. 5d, by changing the permeability of the medium from −0.2 to −0.4, the skin friction coefficient and the Sherwood number were increased while the Nusselt number decreased. As the permeability of the medium changed from −0.2 to −0.4, the skin friction coefficient changed from 1.14173186 to 2.408450783, which shows 110.9471468 % growth in this coefficient, and the Sherwood number changes from 2.603962556 to 2.768844575, which shows a 6.331965820 % growth in this number. Unlike the Sherwood number and the skin friction coefficient, the Nusselt number will decrease from 0.922708144 to 0.903327594, which shows a 2.100398715 % drop in this number. However, according to Figs. (6a-6d), the changes in the positive permeability of the medium are not as effective as the negative permeability of the medium (compare Figs. (5a-5d) with Figs. (6a-6d)). According to Fig. 6a, as the permeability of the medium changes from 0.2 to 0.4, the average velocity decreases from 0.839733015 to 0.832259023, which is a 0.8900438433 % drop in fluid velocity. According to Fig. 6b, as the permeability of the medium changes from 0.2 to 0.4, the average temperature increases from 0.428893896 to 0.431035512, which is a 0.4993346886 % growth in the fluid's temperature. According to Fig. 6c, as the permeability of the medium changes from 0.2 to 0.4, the average concentration increases from 0.15457803 to 0.155232782, which is a 0.4235737769 % growth in the fluid's concentration. According to Fig. 6d, by changing the medium's permeability from 0.2 to 0.4, the skin friction coefficient and the Sherwood number decreased while the Nusselt number increased. As the permeability of the medium changed from 0.2 to 0.4, the skin friction coefficient changed from 4.995998812 to 4.230693156, which shows an 18.08936805 % drop in this coefficient and the Sherwood number changes from 2.937976085 to 2.8994443, which shows a 1.311507782 % drop in this number. Unlike the Sherwood number and the skin friction coefficient, the Nusselt number will increase from 0.869729617 to 0.879223461, which shows a 1.091585685 % growth in this number.

### 4.5 The effect of the magnetic field parameter

**Fig. 7:**
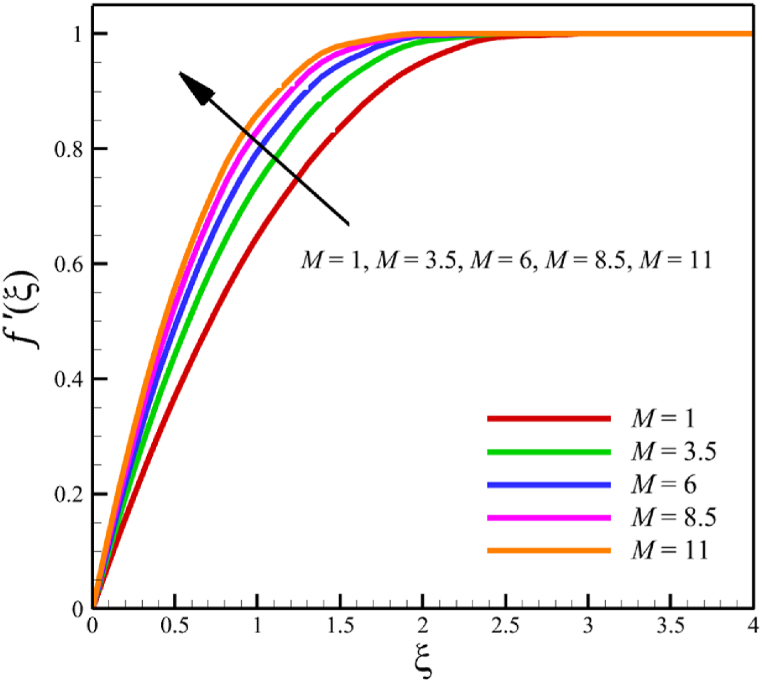

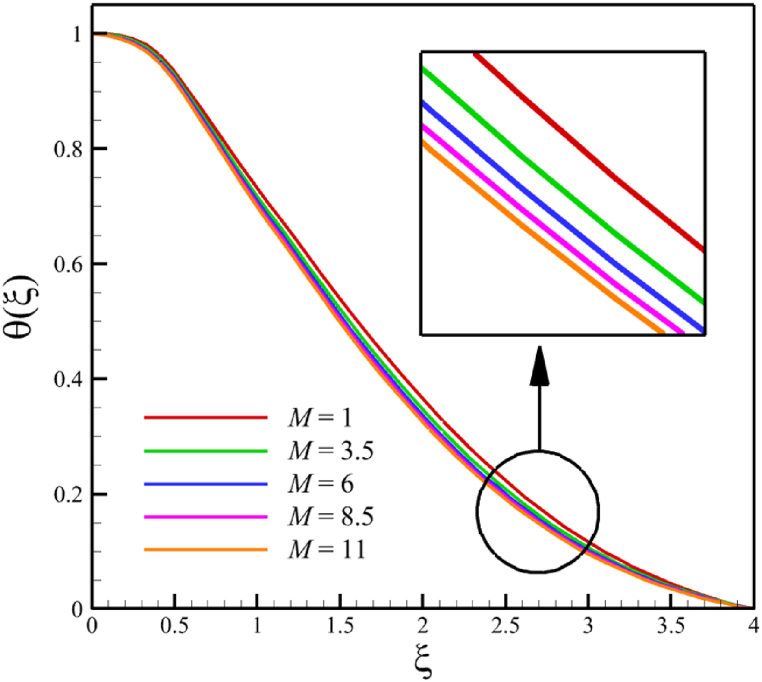

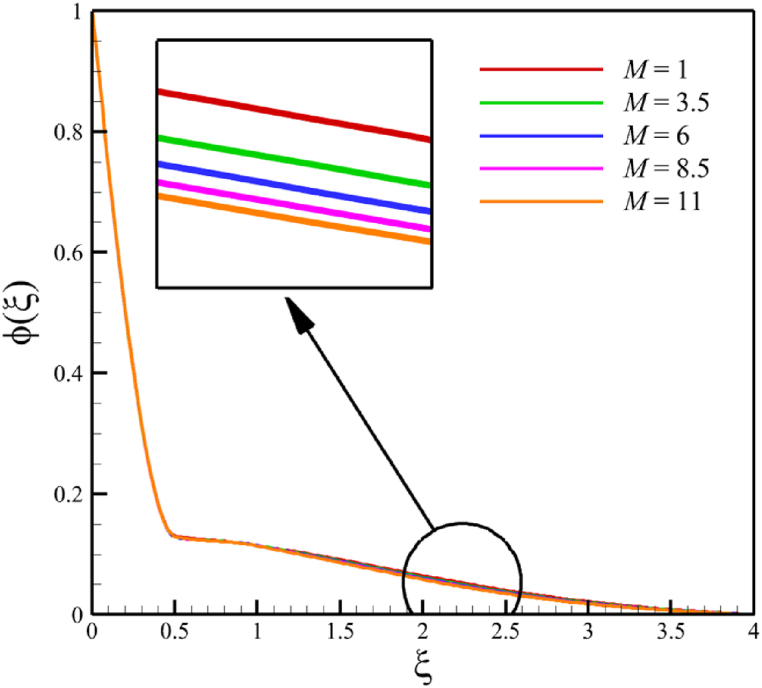

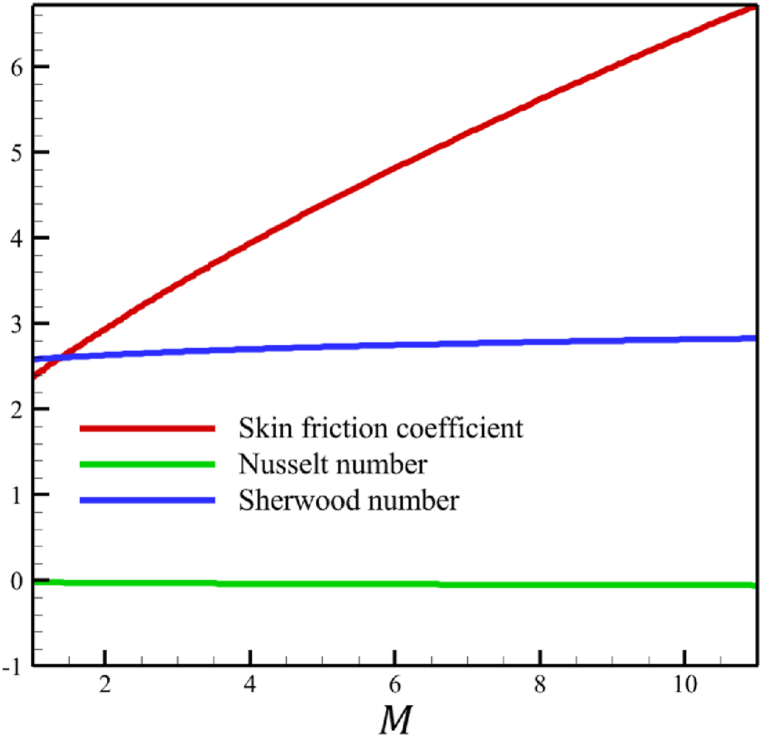
a - The effect on the velocity quantity when the magnetic field parameter changes from 1 to 11.**b -** The effect on the temperature quantity when the magnetic field parameter changes from 1 to 11.**c -** The effect on the concentration quantity when the magnetic field parameter changes from 1 to 11.**d -** The effect on the Nusselt number, skin friction coefficient, and Sherwood number when the magnetic field parameter changes from 1 to 11.

In this subsection, the effect of the magnetic field parameter is investigated. Since the magnetic field parameter has already been defined in Section 2 (Methodology), it has a direct relationship with values such as the electrical conductivity of the nanofluid is denoted with (σ), and the constant of magnetic field perpendicular to the wedge walls ($B_{0}$), while it has an inverse relationship with the density of the base fluid ($\rho_{f}$), and the wedge angle parameter (m). It is obvious that increasing the constant of the magnetic field or choosing a nanofluid with greater electrical conductivity makes the magnetic field parameter larger. However, choosing a base fluid of nanofluid with a higher density can reduce the magnetic parameter's value and the effect of this parameter. Since the value of the wedge angle parameter can only be between zero and one, it does not have much effect on the magnetic parameter compared to the other mentioned parameters, such as the electrical conductivity of the nanofluid, the constant of the magnetic field perpendicular to the wedge walls, and the density of the base fluid. According to Figs. (7a-7d), increasing the magnetic field parameter from 1 to 11 while Wi=8.0, B=2.5, n=0.8, $K_{p}=5.0$, Pr=4.0, Le=3, $D_{4}=0.4$, Sr=0.4, Rd=7, Nb=0.4, Nt=0.4, and m=0.8, will affect the velocity, temperature, concentration, skin friction coefficient, Nusselt number, and Sherwood number. According to Fig. 7a, when the magnetic field parameter changes from 1 to 11, the average fluid's velocity changes from 0.755644128 to 0.821947228, demonstrating the 8.774381689 % growth in the fluid's velocity. According to Fig. 7b, when the magnetic field parameter changes from 1 to 11, the average fluid's temperature changes from 0.43918888 to 0.417886718, demonstrating a 4.850341839 % drop in fluid temperature. According to Fig. 7c, when the magnetic field parameter changes from 1 to 11, the average fluid's concentration changes from 0.163303947 to 0.160635145, demonstrating a 1.634254437 % drop in fluid concentration. According to Fig. 7d, by changing the magnetic field parameter from 1 to 11, the skin friction coefficient and the Sherwood number were increased. As the magnetic field parameter changed from 1 to 11, the skin friction coefficient changed from 2.3820755 to 6.72285096, which shows 182.2266112 % growth in this coefficient, and the Sherwood number changed from 2.585560634 to 2.828490758, which shows a 9.395645989 % growth in this number. The Nusselt number is the same as the Sherwood number because it does not affect significantly when compared with the skin friction coefficient. The Nusselt number enlarged from 0.02038644202 to 0.05556735375 as the magnetic field parameter increased from 1 to 11.

### 4.6 The effect of the wedge angle parameter

**Fig. 8:**
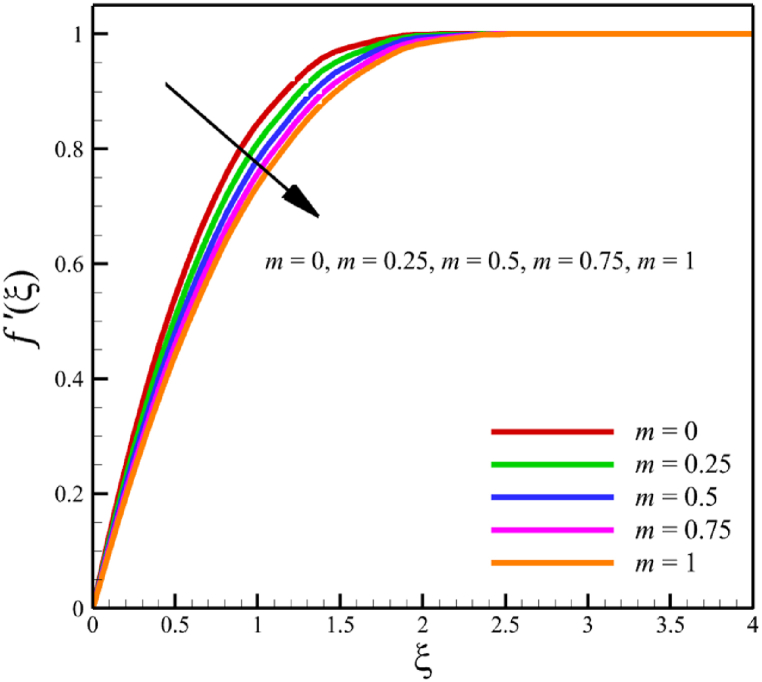

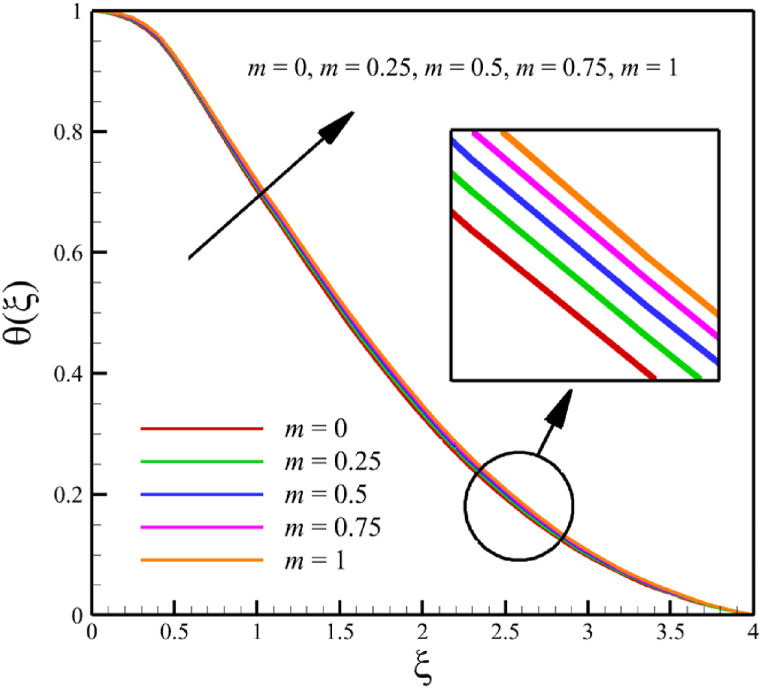

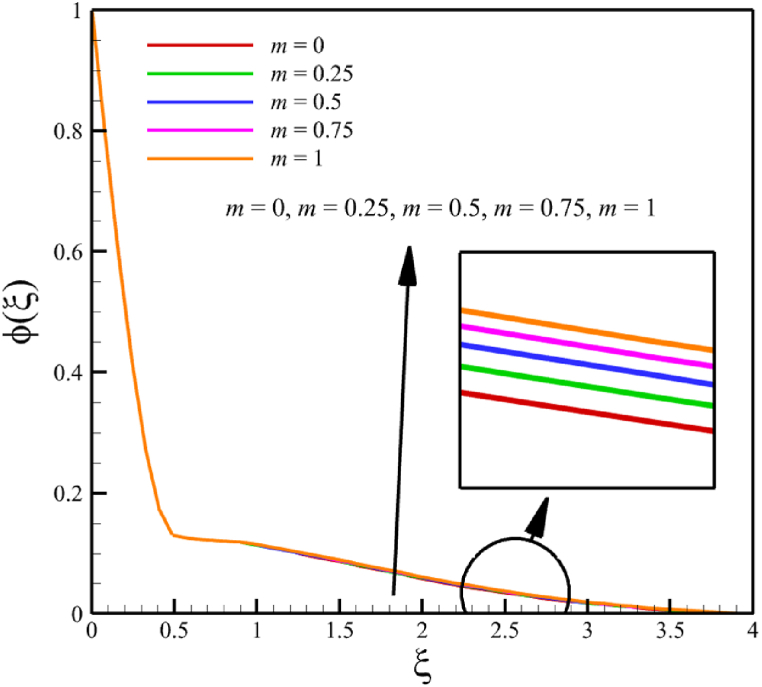

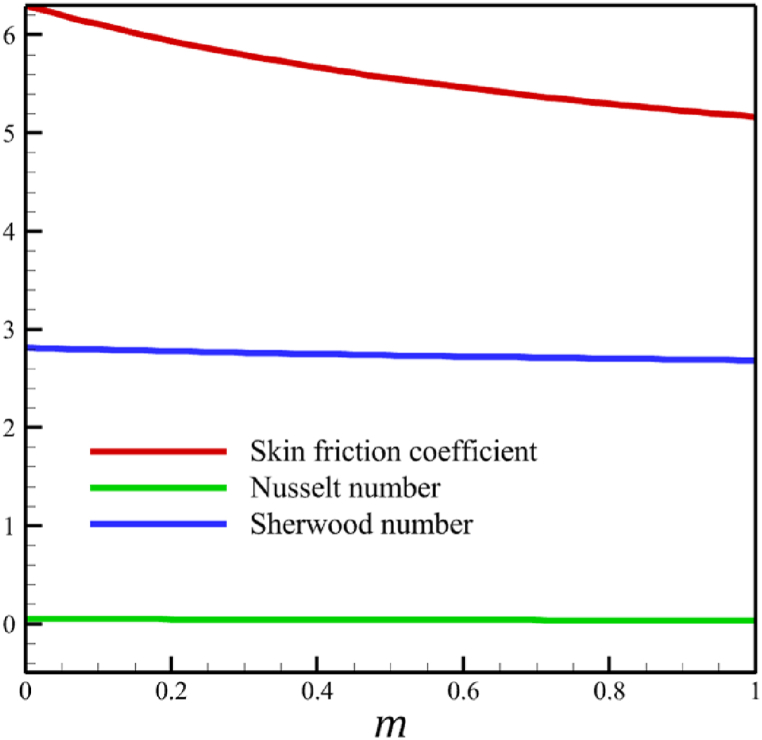
a - The effect on the velocity quantity when the wedge angle parameter changes from 0 to 1.**b -** The effect on the temperature quantity when the wedge angle parameter changes from 0 to 1.**c -** The effect on the concentration quantity when the wedge angle parameter changes from 0 to 1.**d -** The effect on Nusselt number, the skin friction coefficient, and Sherwood number when the wedge angle parameter changes from 0 to 1.

In this subsection, the effect of the wedge angle parameter is investigated. The wedge angle parameter belongs to the geometry of the wedge, and it is limited to values between 0 and 1. As the wedge angle parameter equals 0, the wedge walls are vertical, and when the wedge angle parameter equals 1, the wedge walls are horizontal. The wedge angle parameter itself could directly and indirectly affect the fluid's velocity. The wedge angle parameter's direct effect is due to this parameter's existence in Eq. (21), and indirect effects are related to the influence of wedge angle on parameters such as the Weissenberg number, the permeability of the medium, and the magnetic field parameter. So, for investigating the influence of wedge angle parameters on quantities such as velocity, temperature, concentration, skin friction coefficient, Nusselt number, and Sherwood number, the other parameters such as the Weissenberg number, the permeability of the medium, and the magnetic field parameter that indirectly affected from the wedge angle parameter should be redefined such a way that $Wi=A_{1}\sqrt{\left(m+1\right)}$, $K_{p}=A_{2}\left(m+1\right)$, $M=A_{3}\left(1/\left(m+1\right)\right)$. Here, $A_{1}={\left(\left(\Gamma^{2}{\left(U\left(x\right)\right)}^{3}\right)/\left(\nu x\right)\right)}^{1/2}$, $A_{2}=\left(aK_{0}\right)/\left(2\nu \right)$, and $A_{3}=\left(2\sigma B_{0}^{2}\right)/\left(\rho_{f}a\right)$. According to Figs. (8a-8d), as the wedge angle parameter changes from 0 to 1, the Weissenberg number changes from 8 to 11.31370850, the permeability of the medium changes from 5 to 10, and the magnetic field parameter changes from 11 to 5.5, but other parameters remain unchanged: $A_{1}=8.0$, B=2.5, n=0.8, $A_{2}=5.0$, $A_{3}=11$, Pr=4.0, Le=3, $D_{4}=0.4$, Sr=0.4, Rd=7, Nb=0.4, and Nt=0.4. According to Fig. 8a, as the wedge angle parameter changed from 0 to 1, the average velocity changes from 0.817651102 to 0.785276117, which shows a 3.959510960 % drop in fluid's velocity, while temperature and concentration do not change significantly. The average fluid temperature increased slightly from 0.419337939 to 0.429957294, and the average concentration also grew slightly from 0.16079796 to 0.162066972. The significant rate of change of the dependence of the flow rate on the angle of the wedge and the weak dependence of the concentration and temperature on the angle of the wedge are shown in Fig. 8d.

### 4.7 The effect of the melting heat transfer

**Fig. 9:**
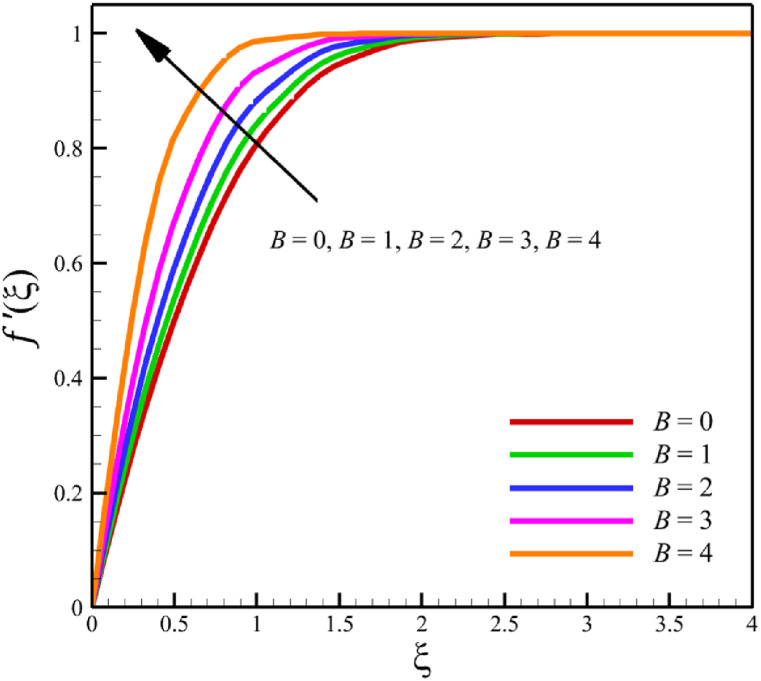

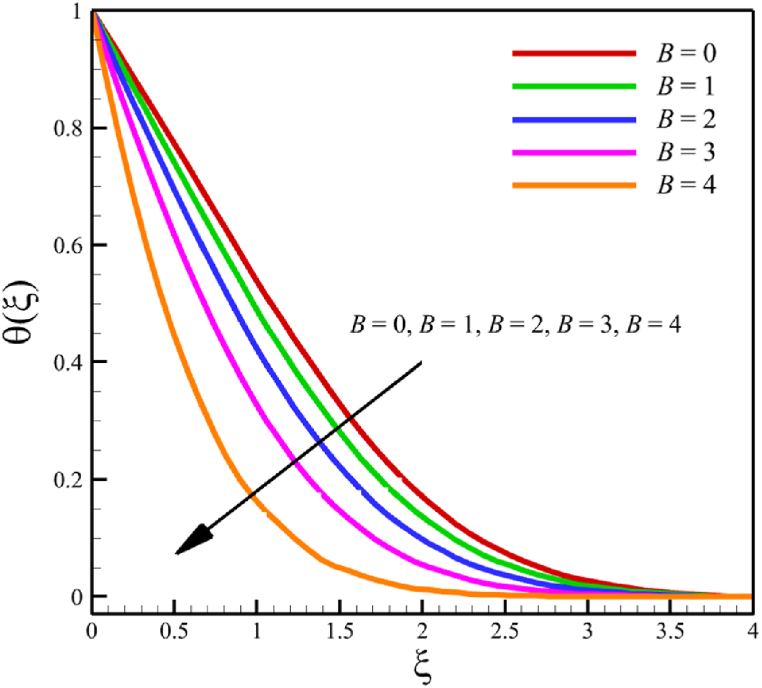

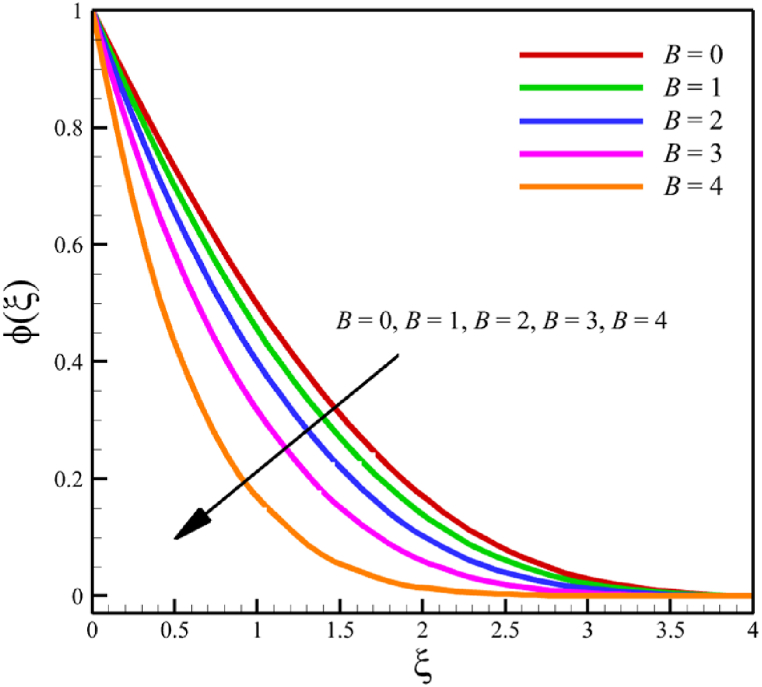

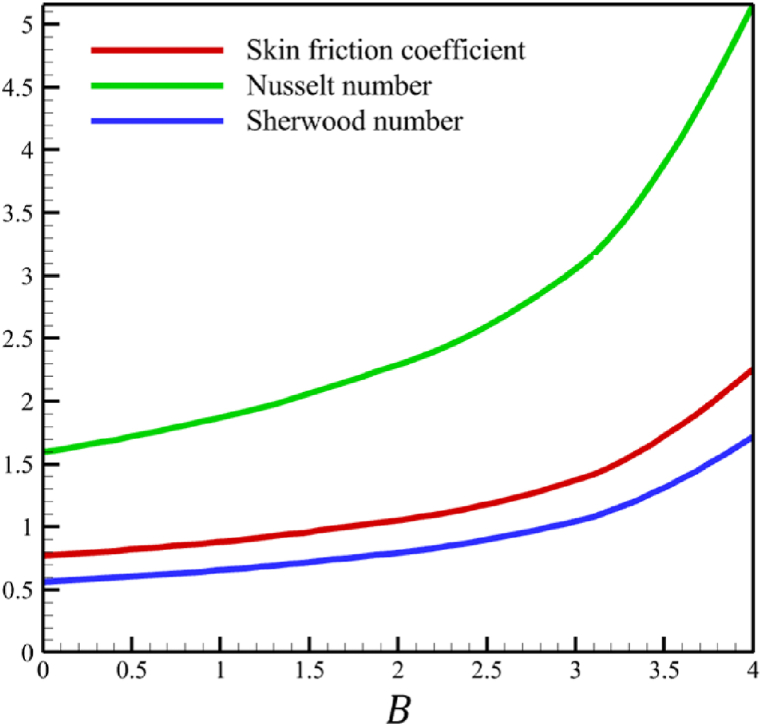
a - The effect on the velocity quantity when the melting heat transfer parameter changes from 0 to 4.**b -** The effect on the temperature quantity when the melting heat transfer parameter changes from 0 to 4.**c -** The effect on the concentration quantity when the melting heat transfer parameter changes from 0 to 4 **d -** The effect on the Nusselt number, the skin friction coefficient, and the Sherwood number when the melting heat transfer parameter changes from 0 to 4.

In this section, the influence of the melting heat transfer parameter is investigated, which is one of the main goals of this research according to the presented physics. The melting heat transfer parameter is located in boundary conditions. So, the melting heat transfer parameter changes significantly affect the fluid's quantities, such as velocity, temperature, concentration, skin friction coefficient, Nusselt number, and Sherwood number. These significant changes can be found in Figs. (9a-9d) where the melting heat transfer parameter changes from 0 to 4, while other parameters remain unchanged: Wi=0.5, n=0.5, M=0.1, $K_{p}=-5.0$, Pr=3.0, Le=0.5, $D_{4}=0.1$, Sr=0.1, Rd=2, Nb=0.2, Nt=0.5, and m=0.5. According to Fig. 9b, the changes in this parameter increase the fluid's average velocity from 0.804876741 to 0.867340672, which showed a 7.760682825 % growth in the fluid's velocity. However, both the fluid's temperature and concertation drop significantly. According to Fig. 9b, the changes of this parameter decreased the average temperature from 0.324503283 to 0.186035306, which showed a 42.67074765 % drop in fluid temperature, and the same thing occurred for the concentration. According to Fig. 9c, the changes in this parameter decrease the average concentration from 0.31412996 to 0.185889434, showing a 40.82403538 % drop in the fluid's concentration.

### 4.8 The effect of the local Reynolds number

**Fig. 10:**
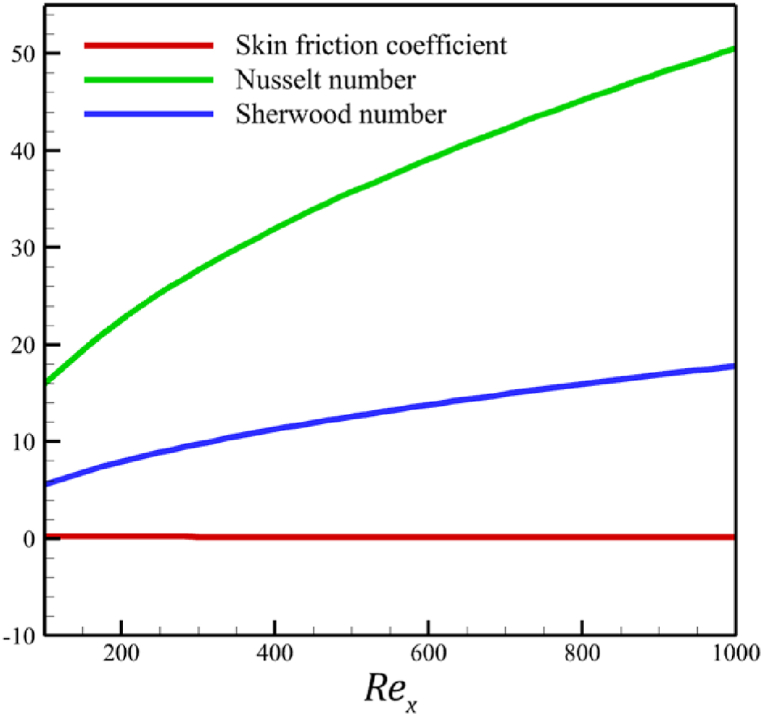
The effect on the Nusselt number, skin friction coefficient, and Sherwood number when the local Reynolds number changes from 0 to 4.

The whole study considers the local Reynolds number so that the fluid regime does not deviate from its laminar flow. Changes in the local Reynolds number will not affect any significant changes in fluid's temperature, velocity, and concentration except for the Nusselt number, skin friction coefficient, and Sherwood number. According to Fig. (10), when the local Reynolds number changes from 100 to 1000, the skin friction coefficient will not change considerably because the skin friction coefficient decreased from 0.236196871 to 0.195543486. However, unlike the skin friction coefficient, the Nusselt and Sherwood number changes are significant. The Nusselt number increased from 15.97857378 to 50.5286869, and the Sherwood number increased from 5.632133963 to 17.81037141.

## 5 Conclusion

### 5.1 Summary of the results

This paper studies the two-dimensional time-independent forced convection, incompressible, MHD flow of the tangent hyperbolic nanofluid on a permeable wedge with heat radiation and melting heat transfer. A suitable similarity transformation was used to reduce the governing equations into a set of nonlinear ODEs with some physical parameters. Among those parameters, only eight were studied due to the aim of the study to consider parameters that were effective on fluid's velocity. The insignificant effects of these parameters on quantities such as temperature and concentration were also investigated, with the addition of insignificant effects on important dimensionless numbers such as Nusselt and Sherwood numbers. Some of these parameters were not investigated due to their ineffective effects on fluid velocity. In addition to novel results, the mathematical method that is applied to solving equations was novel and analytical because the previous similar studies mostly used numerical methods. The analytic solutions were presented and compared with similar studies to validate current results for the special case. Unlike analytic solutions used for validation, the rest of the results with the graphs and graphs were presented quantitatively. In summary, the quantitative results can be concluded qualitatively as follows.

• The fluid velocity drops significantly when parameters such as the Weissenberg number, the power law index, and the wedge angle parameter are increased.
• The fluid velocity grew significantly from increasing the parameters such as the negative permeability of the medium, the magnetic field parameter, and the melting heat transfer parameter.
• The fluid velocity drops insignificantly from increasing the parameters, such as the positive permeability of the medium.
• The fluid temperature and concentration grew insignificantly from increasing the parameters such as the Weissenberg number, the power law index, the positive permeability of the medium, and the wedge angle parameter.
• The fluid temperature and concentration drop insignificantly from increasing the parameters, such as the negative permeability of the medium and the magnetic field parameter.
• As the melting heat transfer parameter increases, the fluid velocity grows significantly; however, unlike fluid velocity, the temperature and concentration will drop significantly.
• The skin friction coefficient exhibits a notably more pronounced increase compared to Nusselt and Sherwood numbers with the escalation of parameters, including the Weissenberg number, power law index, positive and negative permeability of the medium, magnetic field parameter, and wedge angle parameter.
• Increasing the melting heat transfer parameter has a significant effect on increasing fluid velocity, skin friction coefficient, and Nusselt and Sherwood numbers, and at the same time, it brings a significant decrease in temperature and concentration.
• The local Reynolds number significantly affects the Nusselt and Sherwood numbers and, at the same time, does not significantly change the skin friction coefficient.

### 5.2 Suggestions for further study

Many studies [[41], [42], [43], [44], [45], [46], [47]], as it happens today, about fluid flow and heat transfer are still solved numerically, and with the method described in this article, the equations of these studies can also be solved analytically. As observed in the present study, only the parameters that had an effect on the fluid flow rate were investigated, but specifically among these parameters, there was also the melting heat transfer parameter, which, in addition to affecting the fluid flow rate. It also had a significant effect on temperature and concentration. However, other parameters of this study did not significantly affect temperature and concentration. The parameters that affect the temperature and concentration were not investigated because they are far from the main scope of the article, but for further study, the investigation of these parameters can be suggested with the strategy of the present article.

## CRediT authorship contribution statement

**Ali Ahmadi Azar:** Writing – original draft, Software. **Payam Jalili:** Conceptualization. **Zahra Poolaei Moziraji:** Investigation. **Bahram Jalili:** Validation. **Davood Domiri Ganji:** Supervision.

## Declaration of competing interest

The authors declare that they have no known competing financial interests or personal relationships that could have appeared to influence the work reported in this paper.

## References

[1] Choi S.U., Eastman J.A. (1995). Enhancing thermal conductivity of fluids with nanoparticles. Argonne National Lab. (ANL), Argonne, IL (United States).

[2] Mustafa M., Hayat T., Obaidat S. (2012). On heat and mass transfer in the unsteady squeezing flow between parallel plates. Meccanica.

[3] Mahian O. (2013). A review of the applications of nanofluids in solar energy. Int. J. Heat Mass Tran..

[4] Lomascolo M. (2015). Review of heat transfer in nanofluids: conductive, convective and radiative experimental results. Renew. Sustain. Energy Rev..

[5] Ibrahim W. (2017). Magnetohydrodynamics (MHD) flow of a tangent hyperbolic fluid with nanoparticles past a stretching sheet with second order slip and convective boundary condition. Results Phys..

[6] Ali A., Hussain R., Maroof M. (2019). Inclined hydromagnetic impact on tangent hyperbolic fluid flow over a vertical stretched sheet. AIP Adv..

[7] Atif S., Hussain S., Sagheer M. (2019). Effect of viscous dissipation and Joule heating on MHD radiative tangent hyperbolic nanofluid with convective and slip conditions. J. Braz. Soc. Mech. Sci. Eng..

[8] Patil M., Mahesha, Raju C. (2019). Convective conditions and dissipation on Tangent Hyperbolic fluid over a chemically heating exponentially porous sheet. Nonlinear Eng..

[9] Sarkar S., Endalew M.F. (2019). Effects of melting process on the hydromagnetic wedge flow of a Casson nanofluid in a porous medium. Bound. Value Probl..

[10] Endalew M.F., Nayak A., Sarkar S. (2020). FLOW past an oscillating slanted plate under the effects of inclined magnetic field, radiation, chemical REACTION, and TIME-VARYING temperature and concentration. Int. J. Fluid Mech. Res..

[11] Ibrahim W., Gizewu T. (2020). Nonlinear mixed convection flow of a tangent hyperbolic fluid with activation energy. Heat Transfer.

[12] Kebede T. (2020). Heat and mass transfer analysis in unsteady flow of tangent hyperbolic nanofluid over a moving wedge with buoyancy and dissipation effects. Heliyon.

[13] Muhammad R. (2020). Magnetohydrodynamics (MHD) radiated nanomaterial viscous material flow by a curved surface with second order slip and entropy generation. Comput. Methods Progr. Biomed..

[14] Ramaiah K., D (2020). MHD rotating flow of a Maxwell fluid with Arrhenius activation energy and non-Fourier heat flux model. Heat Transfer.

[15] Vaidya H. (2021). Mixed convective nanofluid flow over a non linearly stretched Riga plate. Case Stud. Therm. Eng..

[16] Azeem Khan W. (2022). Impact of time-dependent heat and mass transfer phenomenon for magnetized Sutterby nanofluid flow. Waves Random Complex Media.

[17] Hussain Z., Azeem Khan W. (2022). Impact of thermal-solutal stratifications and activation energy aspects on time-dependent polymer nanoliquid. Waves Random Complex Media.

[18] Tabrez M., Azeem Khan W. (2022). Exploring physical aspects of viscous dissipation and magnetic dipole for ferromagnetic polymer nanofluid flow. Waves Random Complex Media.

[19] Ur Rehman M.I. (2023). Chemical reactive process of unsteady bioconvective magneto Williamson nanofluid flow across wedge with nonlinearly thermal radiation: Darcy–Forchheimer model. Numer. Heat Tran., Part B: Fundamentals.

[20] Israr Ur Rehman M. (2023). "Numerical Analysis of unsteady Nonlinear mixed convection flow of Reiner-Philippoff nanofluid along Falkner-Skan wedge with new mass flux condition.". Chem. Phys. Lett..

[21] Ur Rehman, Chen H., Duraihem F.Z., Hussien M., Hamid A., Qi H. (2024). Darcy-Forchheimer aspect on unsteady bioconvection flow of Reiner-Philippoff nanofluid along a wedge with swimming microorganisms and Arrhenius activation energy. Numerical Heat Transfer, Part A: Applications.

[22] Jalili P. (2022). Heat transfer analysis in cylindrical polar system with magnetic field: a novel hybrid analytical and numerical technique. Case Stud. Therm. Eng..

[23] Jalili Payam, Ali Ahmadi Azar, Bahram Jalili, Davood Domiri Ganji (2023). The HAN method for a thermal analysis of forced non-Newtonian MHD Reiner-Rivlin viscoelastic fluid motion between two disks. Heliyon.

[24] Jalili P. (2023). Study of nonlinear radiative heat transfer with magnetic field for non-Newtonian Casson fluid flow in a porous medium. Results Phys..

[25] Azar E.A. (2023). An exact analytical solution of the Emden–Chandrasekhar equation for self-gravitating isothermal gas spheres in the theory of stellar structures. Physics of the Dark Universe.

[26] Jalili B. (2023). Analytical approach for micropolar fluid flow in a channel with porous walls. Alex. Eng. J..

[27] Jalili P. (2023). A novel technique for solving unsteady three-dimensional brownian motion of a thin film nanofluid flow over a rotating surface. Sci. Rep..

[28] Jalili P. (2023). A novel analytical Investigation of a swirling fluid Flow and a rotating Disk in the Presence of uniform suction. Arabian J. Sci. Eng..

[29] Jalili B. (2023). Impact of variable viscosity on asymmetric fluid flow through the expanding/contracting porous channel: a thermal analysis. Case Stud. Therm. Eng..

[30] Endalew M.F., Sarkar S. (2023). Numerical exploration of forced convection hydromagnetic hyperbolic tangent nanofluid flow over a permeable wedge with melting heat transfer. Sci. Rep..

[31] Ishak A., Nazar R., Pop I. (2008). MHD boundary-layer flow of a micropolar fluid past a wedge with variable wall temperature. Acta Mech..

[32] Li Q., Zhu G. (2021). Controlling negative permittivity and permeability behavior in LaFeO3 through sintering temperature. Ceram. Int..

[33] Tsutaoka T. (2013). Negative permittivity and permeability spectra of Cu/yttrium iron garnet hybrid granular composite materials in the microwave frequency range. Appl. Phys. Lett..

[34] Nadeem S., Akram S. (2011). Magnetohydrodynamic peristaltic flow of a hyperbolic tangent fluid in a vertical asymmetric channel with heat transfer. Acta Mech. Sin..

[35] Akbar N.S. (2013). Numerical solutions of magnetohydrodynamic boundary layer flow of tangent hyperbolic fluid towards a stretching sheet. Indian J. Phys..

[36] Nadeem S., Shahzadi I. (2016). Inspiration of induced magnetic field on nano hyperbolic tangent fluid in a curved channel. AIP Adv..

[37] Hayat T. (2017). Stagnation point flow of hyperbolic tangent fluid with Soret-Dufour effects. Results Phys..

[38] Ganesh Kumar K., Gireesha B., Gorla R. (2018). Flow and heat transfer of dusty hyperbolic tangent fluid over a stretching sheet in the presence of thermal radiation and magnetic field. Int. J. Mech. Mater. Eng..

[39] Ahmadi Azar A. (2023). "Investigating the effect of structural changes of two stretching disks on the dynamics of the MHD model.". Sci. Rep..

[40] Jalili B. (2024). "A novel approach to micropolar fluid flow between a non-porous disk and a porous disk with slip.". Chin. J. Phys..

[41] Rehman M.I.U. (2022). Thermal radiative flux and energy of Arrhenius evaluation on stagnating point flowing of Carreau nanofluid: a thermal case study. Case Stud. Therm. Eng..

[42] Israr Ur Rehman M., Chen H., Hamid A., Jamshed W., Eid M.R., Duraihem F.Z., Alqahtani H. (2023). Thermal analysis of radiative and electromagnetic flowing of hybridity nanofluid via Darcy–Forchheimer porous material with slippage constraints. Energy & Environment.

[43] Rehman M.I.U. (2023). "Effect of Cattaneo-Christov heat flux case on Darcy-Forchheimer flowing of Sutterby nanofluid with chemical reactive and thermal radiative impacts.". Case Stud. Therm. Eng..

[44] Rehman M.I.U., Chen H., Hamid A. (2022). Theoretical investigation of Darcy-Forchheimer flow of bioconvection Casson fluid in the presence of chemical reaction effect. Biomass Conv. Bioref..

[45] Rehman M.I.U. (2024). "Analysis of Cattaneo–Christov heat flux and thermal radiation on Darcy–Forchheimer flow of Reiner–Philippoff fluid.". Int. J. Mod. Phys. B.

[46] Rehman M.I.U. (2024). "Impact of bioconvection on Darcy-Forchheimer flow of MHD Carreau fluid with Arrhenius activation energy.". ZAMM-Journal of Applied Mathematics and Mechanics/Zeitschrift für Angewandte Mathematik und Mechanik.

[47] Rehman M.I.U. (2023). "Thermal and solutal slip impacts of tribological coatings on the flow and heat transfer of reiner-philippoff nanofluid lubrication toward a stretching surface: the applications of Darcy-Forchheimer theory.". Tribol. Int..

